# The effects of aging on molecular modulators of human embryo implantation

**DOI:** 10.1016/j.isci.2021.102751

**Published:** 2021-06-19

**Authors:** Panagiotis Ntostis, Grace Swanson, Georgia Kokkali, David Iles, John Huntriss, Agni Pantou, Maria Tzetis, Konstantinos Pantos, Helen M. Picton, Stephen A. Krawetz, David Miller

**Affiliations:** 1 Discovery and Translational Science Department, Leeds Institute of Cardiovascular and Metabolic Medicine, University of Leeds, Leeds, LS2 9JT, UK; 2 Genetics Department, Medical school, National and Kapodistrian University of Athens, Athens, 115 27, Greece; 3 Department of Obstetrics and Gynecology and the Center for Molecular Medicine and Genetics, Wayne State University School of Medicine, Detroit, MI 48201, USA; 4 Genesis Athens Clinic, Reproductive Medicine Unit, Athens, 152 32, Greece

**Keywords:** Embryology, Omics

## Abstract

Advancing age has a negative impact on female fertility. As implantation rates decline during the normal maternal life course, age-related, embryonic factors are altered and our inability to monitor these factors in an unbiased genome-wide manner *in vivo* has severely limited our understanding of early human embryo development and implantation. Our high-throughput methodology uses trophectoderm samples representing the full spectrum of maternal reproductive ages with embryo implantation potential examined in relation to trophectoderm transcriptome dynamics and reproductive maternal age. Potential embryo-endometrial interactions were tested using trophectoderm sampled from young women, with the receptive uterine environment representing the most ‘fertile’ environment for successful embryo implantation. Potential roles for extracellular exosomes, embryonic metabolism and regulation of apoptosis were revealed. These biomarkers are consistent with embryo-endometrial crosstalk/developmental competency, serving as a mediator for successful implantation. Our data opens the door to developing a diagnostic test for predicting implantation success in women undergoing fertility treatment.

## Highlights

• Trophectoderm transcriptome profiles are altered with maternal age
• Trophectoderm transcriptome profiles can delineate maternal reproductive biological age
• A genome-wide methodology reveals potential embryo-endometrial communication
• Exosome-related expression that could mediate implantation success decreases with aging

## Graphical abstract

**Figure:**
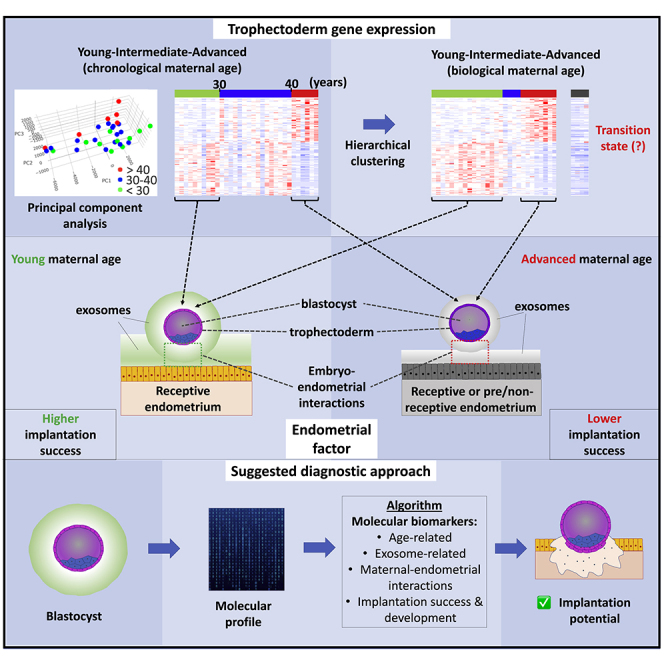

## Introduction

In recent years, maternal age, which heavily influences reproductive success rates, has been rising worldwide (Schmidt et al., 2012). Indeed, maternal age is one of the strongest predictors for assisted reproductive technology (ART) success (Nelson and Lawlor, 2011). Younger women (<35 years old) generally report higher implantation, pregnancy, and delivery rates (Practice Committee of the American Society for Reproductive Medicine, 2006; Hull et al., 1996). Decreased live birth rates are correspondingly observed in women over 35 years, even when euploid embryos are transferred during ART procedures (Scott et al., 2012). Implantation and pregnancy rates are reduced for women over the age of 40 (Harton et al., 2013). These results suggest that in addition to the known risk of increased developmentally lethal aneuploidy (Irani et al., 2019), other poorly understood age-related factors must impact implantation and inevitably, pregnancy.

Euploid blastocyst uterine transfer yields implantation rates of between 50% and 80% and unexplained/idiopathic infertility accounts for 14–26% of cases of implantation failure (Saravelos and Li, 2012). Abnormal patterns of embryonic gene expression associated with maternal aging may partially explain these failures with unsuccessful embryo development and implantation reflecting a change in the relative abundance of factors present in the less fertile aging reproductive population (Harton et al., 2013; Lee et al., 2014; Scott et al., 2012). In humans, major embryonic genome activation (EGA) events generally occur during the third day (post-fertilization) and when mistimed, can compromise embryo growth (Niakan et al., 2012). The trophectoderm is the first embryonic cell layer to make contact with the uterine endometrium, playing a vital role in embryo-endometrial communication and decidual invasion. Trophectoderm cells, like other stem cells, exhibit similar aging processes that distinguish them from more highly differentiated cells (Giakoumopoulos and Golos, 2013; Marikawa and Alarcón, 2009; Roberts and Fisher, 2011; Tam et al., 2007). Together with post-transcriptional and post-translational modifications, the embryonic genome regulates the trophectoderm transcriptome which may in turn impact implantation and live birth rates (Jones et al., 2008; Li et al., 2017; Scott et al., 2012; Harton et al., 2013; Reyes and Ross, 2016; Winata and Korzh, 2018; Vastenhouw et al., 2010; Cimadomo et al., 2018; Ntostis et al., 2019; Parks et al., 2011).

Stem cell lineages of younger and older animals can be distinguished by changes in the expression of exosomal biomarkers, supporting the view that exosomal payloads and ‘signatures’ are altered by aging (Lei et al., 2017). Blastocyst-secreted exosomes may affect endometrial receptivity and gene expression, potentially influencing implantation and the establishment of pregnancy (Simón et al., 2000; Desrochers et al., 2016; Giacomini et al., 2017; Saadeldin et al., 2015; Greening et al., 2016; Homer et al., 2017). In this regard, trophectoderm cells from the embryos of older and younger women may have distinctive exosomal ‘signatures’ that predict the likelihood of implantation success. To test this hypothesis, we examined the effect of maternal age on trophectoderm transcriptome dynamics by modeling human embryo-endometrial interactions as a function of reproductive age, focusing on the most ‘fertile’ state of the embryo (young maternal age or YMA, 20–29 years). We uncovered a set of molecular biomarkers that could predict the most competent blastocyst for embryo transfer. This system enabled insight into the reproductive transition period for women of intermediate maternal age (IMA, 30–39 years), highlighting potential benefits for the clinical management of older infertile patients.

## Results

### 1. Clinical samples

**Figure 1:**
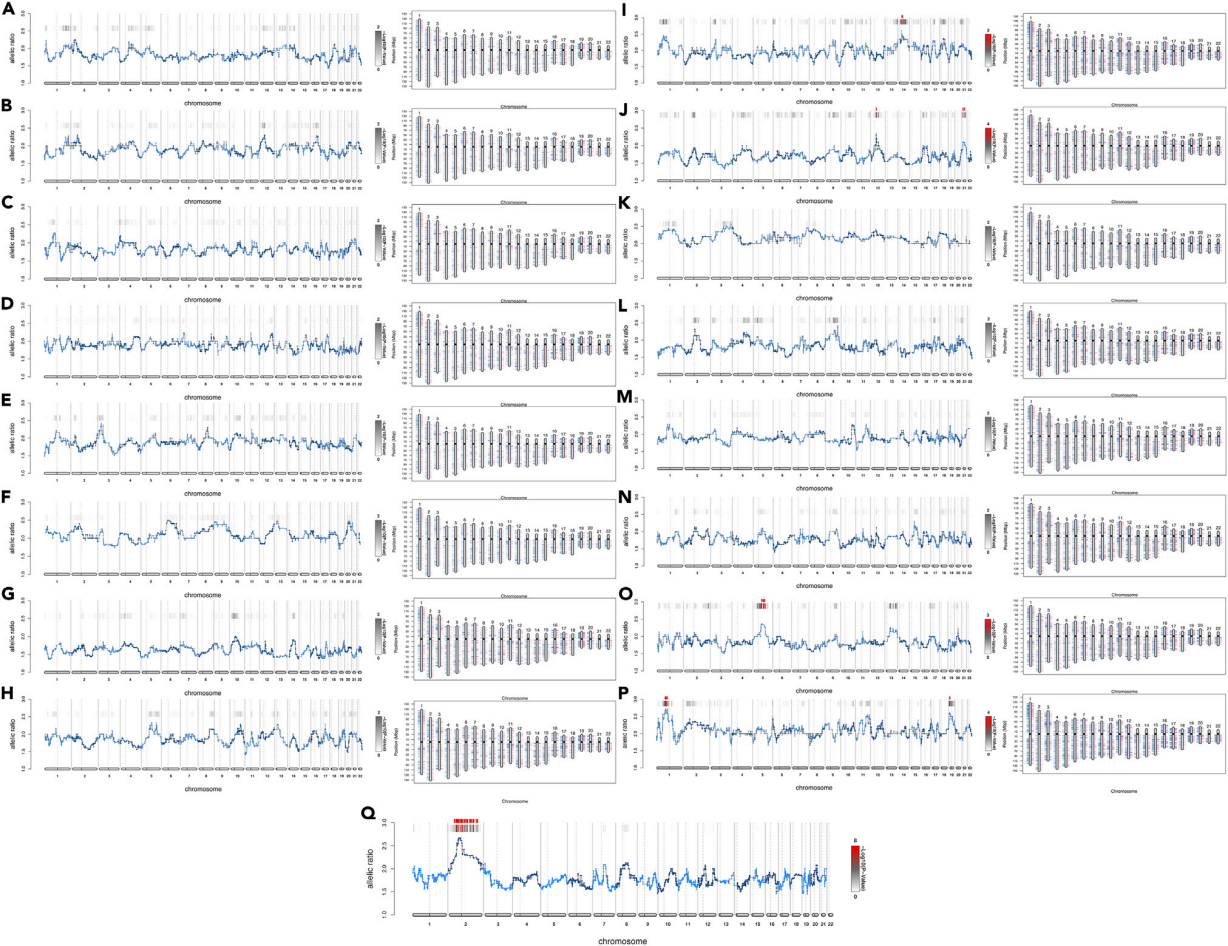
Expressed-SNP-karyotyping (eSNP-karyotyping) analysis on the trophectoderm RNA sequencing data (A–P) Allelic ratio (left side) and loss of heterozygosity (LOH) (right side) per plot pair were reported for each blastocyst derived from the YMA (A-J) and AMA (K-P) cohorts. Homozygous SNPs are shown in blue and present or absent heterozygous SNPs are shown in pink and red respectively for each autosome, indicating that fully representative SNP coverage was obtained for all biopsies. The scale to the right of the allelic ratio diagrams represents –log(10) p values using false discovery rate (FDR) correction for multiple testing. The darker shades shown for the bars above the traces correspond to increasing fold change. The software flags the possibility for duplication in red. Both the allelic ratios and LOH maps are used to indicate whether an abnormality is likely to be a duplication or deletion. Apart from the 10^th^ sample (J) where the duplication seems to extend across the whole chromosome, suggesting trisomy 21, the remaining red flags covered only peri-centromeric regions, which are not related to whole chromosome aneuploidies (detailed explanations in the STAR Methods section: Aneuploidy detection using RNA sequencing data). (Q) Example of a trophectoderm sample with a trisomy of chromosome 2. Allelic ratio plots (left) were reported for each blastocyst derived from a trisomic sample. Homozygous SNPs are shown in blue and heterozygous SNPs shown were present (pink) or absent (red), for each autosome, indicating that fully representative SNP coverage was obtained. The scale to the right of the allelic ratio diagrams represents –log(10) p values following correction for multiple testing using FDR. The bars with differential shading above the traces indicate that darker shades correspond to increasing fold changes. The software flags the possibility of duplication in red.

Gene expression profiles were generated from trophectoderm biopsies obtained from a total of 36 individual Day 5 blastocysts following informed consent. Expressed-SNP-karyotyping (eSNP-karyotyping) analysis of the trophectoderm deep sequencing data (Weissbein et al., 2016; Ntostis et al., 2019), excluded samples with aneuploidies indicative of a non-viable embryo from further consideration, with a total of 32 samples passing all inclusion criteria (Figure 1). These samples were from four women aged 30 years and younger (YMA cohort), eight women between 30 and 39 years old (IMA cohort) and three women aged 40 and older (advanced maternal age - AMA cohort). Ten trophectoderm biopsies were obtained from the YMA, sixteen from the IMA, and six from the AMA cohort, with an average respective maternal age per blastocyst of 24.4 ± 2.0 years, 34.3 ± 2.6 years and 42 ± 1.1 years (mean ± SD). No statistically significant differences were found in the corresponding paternal age groups with mean age and standard deviation (mean ± SD) in the YMA, IMA, and AMA cohorts being 43.6 ± 5.7 years, 40.3 ± 6.5 years, and 41.3 ± 2.5 years, respectively. The distribution of the embryonic developmental stages among the three maternal age cohorts were similar, indicating that maternal age was the main or sole factor affecting trophectoderm gene expression.

### 2. Identifying the relationship between maternal age and trophectoderm gene expression

Following differential expression (DE) analyses, a total of 234 protein-coding gene transcripts were shared between YMA vs AMA and IMA vs AMA comparisons, with 346 transcripts differentially expressed in the YMA vs AMA comparison (Table S1). No DE transcripts were detected when the YMA and IMA cohorts were compared. Comparison of YMA and AMA cohorts returned 580 DE transcripts (or 233 unique genes) in total. These correspond to 312 (111 genes) and 268 (122 genes) transcripts that were more highly expressed in the YMA and AMA cohorts, respectively (Table S2).

**Figure 2:**
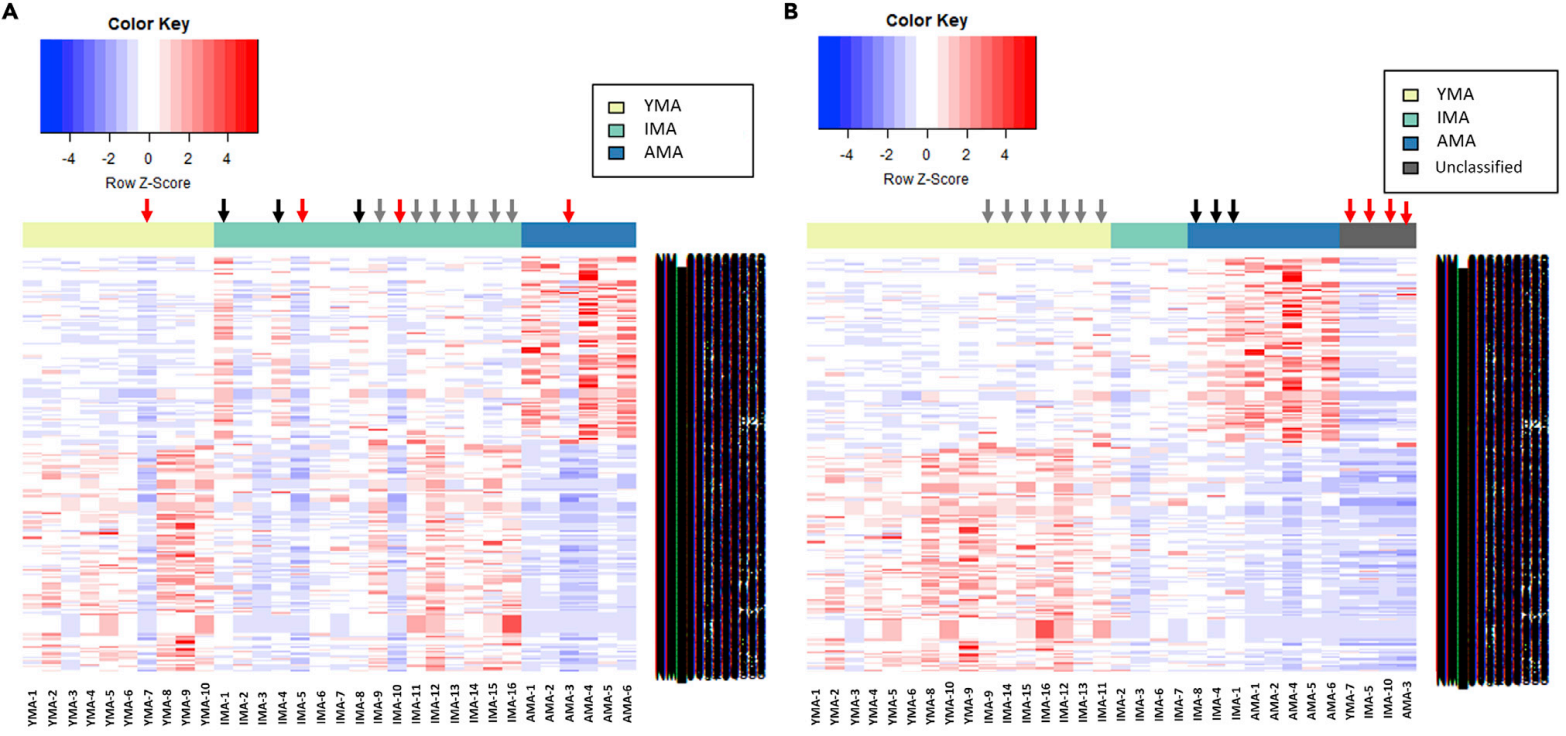
Heatmap of differential expression (DE) genes in YMA vs AMA and IMA vs AMA comparisons (A) Samples ordered by chronological maternal age (YMA cohort = yellow, IMA cohort = green, AMA cohort = blue). (B) Samples ordered by biological maternal reproductive age (rba-YMA cohort = yellow, rba-IMA cohort = green, rba-AMA cohort = blue) and samples that were called as ‘unclassified’ (gray) due to their unique gene expression patterns. Black arrows indicate samples that behave more like the AMA samples and gray arrows indicate samples that behave like the YMA samples. Red arrows illustrate samples that become part of the ‘unclassified’ group when considered by biological reproductive age. Gene expression levels are illustrated as Z-scores (blue to red). The black bars to the right of the heatmaps indicate 632 gene names, which are too densely packed to be legible here. The full list is available in Tables S1 and S2.

The clustered heatmap based on chronological maternal age placed the YMA, IMA, and AMA cohorts into three separate groups (Figure 2). Gene expression patterns of IMA samples showed either similarities with the YMA or AMA transcriptomes or a distinctive gene expression profile. The YMA and AMA samples, however, always retained distinct gene expression patterns (Figure 2A), suggesting that there was a clear correspondence between the YMA/AMA trophectoderm gene expression patterns and maternal age. Using this as a guide, hierarchical clustering analysis on the differentially expressed trophectoderm genes was performed as a function of chronological age across the YMA and AMA datasets, guiding the reproductive biological age (rba) classification of the IMA samples. In this regard, hierarchical clustering analysis of trophectoderm gene expression patterns separating the rba groups, were generated independent of the chronological maternal age. Differential gene expression patterns have been employed previously to determine biological age in different cell types (Jylhävä et al., 2017; Holly et al., 2013; Shavlakadze et al., 2019; Lin et al., 2019a). These studies suggest that as aging affects the transcriptomes of different cell types, the transcriptome could be a useful indicator/predictor of maternal biological age irrespective of chronological age. In this context, the current study distinguished four biological age patterns based on the hierarchical clustering of trophectoderm gene expression among the samples (Figure 2B).

The changes in sample classification from chronological to biological maternal age are best illustrated by the IMA samples, as indicated by the colored arrows in the transition from the clustering by chronological age (Figure 2A) to the clustering based on the rba (Figure 2). The rba-IMA cohort displayed a unique pattern, suggesting that a biological age determinant based on the gene expression patterns, rather than chronological age clustering, better described the cohort. For example, all IMA samples indicated by gray arrows (IMA-9, IMA-11, IMA-12, IMA-13, IMA-14, IMA-15, IMA-16), clustered within the YMA cohort, whereas all IMA samples indicated by black arrows (IMA-1, IMA-4, IMA-8) clustered within the AMA cohort. The samples identified by red arrows (YMA-7, IMA-5, IMA-10, AMA-3) may lie between the rba-IMA and rba-AMA cohorts, although herein they are clustered as ‘unclassified’ due to their unique gene expression pattern (Figure 2B).

**Figure 3:**
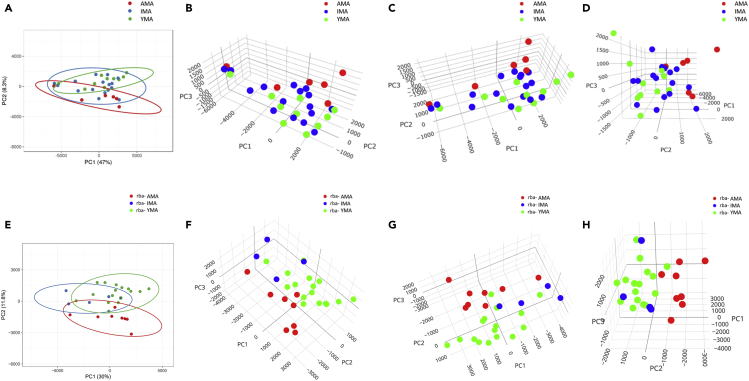
Singular value decomposition (SVD) plot representing the trophectoderm transcriptome from YMA/rba-YMA (green), IMA/rba-IMA (blue) and AMA/rba-AMA (red) cohorts (A) Visualization of chronological age samples is based on the first two Principal Components (PC1, PC2). (B–D) Three-dimensional visualization of three separate views of the chronological age samples, based on the first three PCs (PC1, PC2, PC3). Pareto scaling was applied to SVD with imputation for principal component calculations. (E) Visualization of biological age samples, based on the first two Principal Components (PC1, PC2). (F–H) Three-dimensional visualization of three separate views of the biological age samples (rba-YMA, rba-IMA, rba-AMA), based on the first three PCs (PC1, PC2, PC3). Pareto scaling was applied to SVD with imputation for principal component calculations.

Further insights into the trophectoderm transcriptome relationships were revealed by principal component analysis (PCA). When trophectoderm samples were clustered based on the chronological age, a gradual shift of the trophectoderm transcriptome from YMA to IMA to AMA (Figures 3A–3D) was seen, with the YMA and AMA cohorts lying the furthest apart. IMA samples were less homogeneous than YMA and AMA. As expected, following clustering by reproductive biological age, PCA showed clear differences between rba-YMA and rba-AMA cohorts with the rba-IMA samples lying closer to the rba-YMA cohort (Figures 3E–3H). Comparison between the rba-YMA and rba-AMA cohorts revealed 1,129 DE transcripts with 655 being significantly upregulated in the rba-YMA cohort (Table S3). The statistical variability was approximately 0.45 (cpm>1), reducing to 0.39 (cpm>5) when only the YMA and AMA cohorts were considered. A small increase of ∼9% in the number of transcripts was reported when the cpm threshold was reduced to 1. This is consistent with a study powered to 0.7. Comparing the DE genes according to the biological versus chronological age yielded a 75% overlap in the YMA gene transcripts compared to a 70% overlap in the total DE gene transcripts between YMA and AMA cohorts. Overall, consistent gene expression patterns between YMA/rba-YMA and AMA/rba-AMA cohorts were apparent (Figures S1 and 2), strengthening these observations.

### 3. Embryo implantation success with respect to reproductive maternal age

The implantation rates for the transferred blastocysts were determined by elevated serum beta-human chorionic gonadotropin (beta-hCG) levels and the appearance of a fetal sac or fetal heartbeat by ultrasound scan. All transferred rba-YMA blastocysts implanted (n = 13/13), while only half of the rba-AMA blastocysts implanted (n = 4/8) and this is reflected in the clustering of the trophectoderm samples by reproductive biological age. A statistically significant difference (two-tailed Fisher’s test, p = 0.012) was observed, supporting the hypothesis that factors expressed in the YMA trophectoderm are important for successful embryo implantation in the human. In this regard, both the trophectoderm gene expression patterns and the implantation success support the efficacy of the classification by reproductive biological age.

### 4. Functional annotation of the trophectoderm transcriptome during aging and candidate molecular biomarkers for implantation success

*Ontological* analysis was undertaken to provide an overview of the biological mechanism underpinning the implantation process (Tables S2 andS3). Following on from the DE analysis between rba-YMA and rba-AMA samples, enrichment for functional annotation was assessed among the more highly expressed genes in each group. The top biological processes for rba-YMA trophectoderm included *isoprenoid* (FDR 1.4E-3)*, cholesterol* (FDR 3.1E-12) *and lipid biosynthetic processes,* similar to the YMA cohort, along with genes involved in mitochondrial ATP synthesis (FDR 4.8E-4 to 1.7E-2) and extracellular exosome biogenesis and function (Table S4). *Cell-cell adhesion* also appeared to be well represented in the rba-YMA cohorts, while the rba-AMA cohort reported no significant enrichment for any ontologies (Table S4). Interestingly, cellular component analysis on the YMA cohort revealed strong association with *extracellular exosomes* based on chronological age (FDR 8.2E-06) that further increased as a function of reproductive biological age (FDR 1.7E-17) (Table S4). Μitochondrial-related cellular components, such as *mitochondrial inner membrane* and *ATP-synthase*, were significantly higher in the rba-YMA cohort, with no significant ontologies found in the rba-AMA cohort. This suggests a novel route for successful embryo-endometrial communication, involving an age-related subset of human exosomal embryonic genes as reported herein.

The ontological results following differential gene expression analysis of deep sequencing data provided candidate trophectoderm genes (molecular biomarkers) for subsequent validation. Genes reported in the exosome-related ontologies included *DAB2*, *DBI, FABP3*, and *IFITM3* and were investigated for enrichment in the YMA/rba-YMA cohorts. The AMA/rba-AMA cohort genes were mildly enriched in processes potentially integral to blastocyst degeneration. *BAK1*, *BRAP*, and *ZFAND5* genes were selected for their association with apoptosis and ubiquitination, and *CCPG1* for the regulation of cell cycle. Consistent differential gene expression patterns between the YMA/rba-YMA and AMA/rba-AMA cohorts were revealed by both RNA sequencing and RT-qPCR approaches with *DAB2, FABP3*, *and IFITM3* showing approximately 6, 15, and 14 times higher levels of gene expression, respectively, in the YMA/rba-YMA than in the AMA/rba-AMA cohorts. Compared with the YMA/rba-YMA cohort, *BAK1, ZFAND5*, and *BRAP* gene expression levels were, respectively, approximately 6, 3, and 2.5 times higher in the AMA/rba-AMA cohorts (Figure S1).

### 5. Revealing candidate trophectoderm molecular biomarkers potentially involved in maternal-fetal interactions

**Figure 4:**
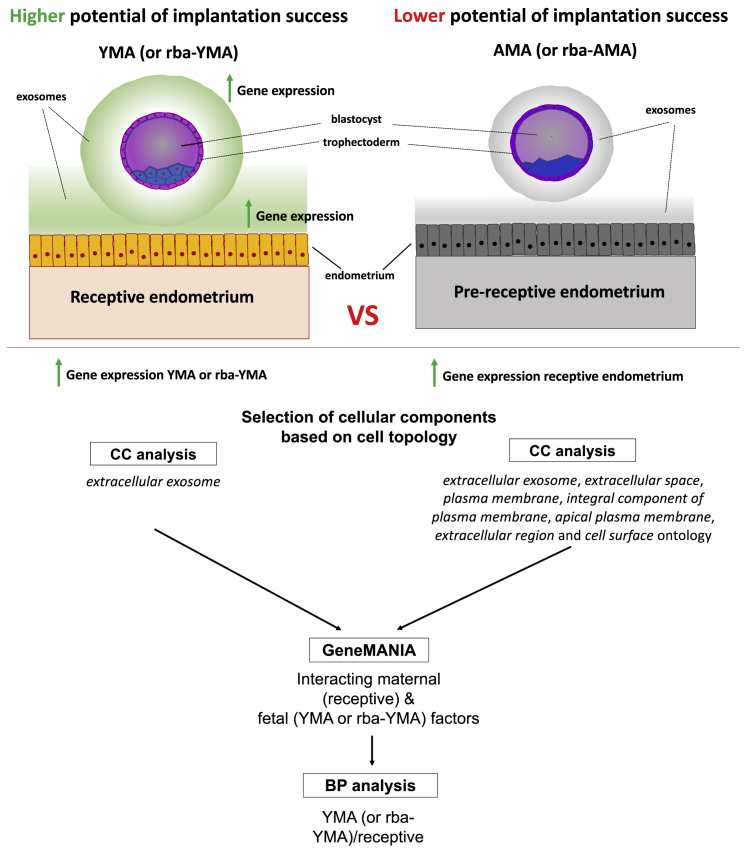
Maternal-fetal interaction model Pipeline used to assess trophectoderm transcriptomes from YMA and AMA cohorts together with endometrial receptive and pre-receptive RNA sequencing data. In brief, differential gene expression was followed by cellular component (CC) analysis on the trophectoderm transcriptome. Ontologies that could support maternal-fetal communication were selected. Statistically significantly more highly expressed exosomal genes were used in the downstream analysis using the Cytoscape GeneMANIA module. A similar approach was applied to endometrial genes (receptive and pre-receptive cohorts), where exosome and plasma membrane-related ontologies from receptive endometrial cohorts together with the trophectoderm gene lists derived from YMA women, were subjected to GeneMANIA alongside the interacting factors to biological process (BP) analysis.

Constitutive maternal factors of receptive endometrial cells and their potential interactions with a series of YMA/rba-YMA trophectodermal *extracellular exosome* factors were key findings of the study. To understand the effect of transcriptomic dynamics on embryo-endometrial communication, we developed an *in silico* molecular maternal-fetal interaction model in the context of the receptive endometrium (Figure 4). This model relied initially on the cellular components involving maternal factors and their interaction with a series of YMA/rba-YMA trophectodermal *extracellular exosome* and *cell adhesion* molecules. These maternal factors are involved in cellular components including *cell surface*, *extracellular exosome*, *extracellular space*, and *extracellular region*, along with the *plasma membrane*-related cellular components (Figure 4). Cytoscape GeneMANIA classified these trophectoderm and endometrial factors based on their physical interactions and shared common pathways (Figures S2 and S3), increasing the probability for actual embryo-endometrial communications (full Cytoscape networks are available on request). This process revealed 133 (chronological age analysis) and 253 (reproductive biological age analysis) potentially interacting trophectoderm-endometrial gene products (Table S5) that went through ontological analysis to reveal the biological processes shared between the trophectoderm and endometrial factors in the event of close proximity.

The biological process analysis considered both the embryonic and endometrial factors. Focusing on the biological processes that included at least 3 embryonically derived factors, trophectoderm *DAB2*, *PRDX2*, *UBB*, and *WNT7A*, were among the genes classified as enriched for *negative regulation of apoptotic process* (FDR 9.5E-4), together with 13 endometrial expressed genes, including *SMAD3*, *PIK3R1*, and *PSEN1* (Table S6). Apoptosis is a crucial process during embryo implantation (Boeddeker and Hess, 2015). Enrichment in the *MAPK* cascade included the trophectoderm transcripts *PEBP1*, *PSMA5*, and *UBB*, accompanied by 8 endometrial genes including *FYN* and *GRB2* (Table S6). Additional biological processes indicative of a receptive endometrium alongside rba-YMA factors also revealed significant enrichment for *cell-cell adhesion* (FDR 1.5E-6), *signal transduction* (FDR 1.1E-3) and *positive regulation of cell migration* (FDR 1.3E-3) (Table S6). Embryonically expressed genes, including DAB2, CD274, and HSP90AA1, participate in signal transduction and the positive regulation of cell migration together with other maternal genes (Table S6). *HSPB11* and *HSP90AA1* represent important molecular chaperones highly expressed in the YMA/rba-YMA trophectoderm cohorts with potential function in embryonic implantation. PRDX2, HSP90AA1, and PSMA5 are of particular interest, as they appear to be present at the protein level in the extracellular space of human blastocysts (Poli et al., 2015). This cluster of interactions suggests that when in close proximity, maternal and fetal molecules may finely tune cell-signaling processes to regulate apoptosis, indicating that the associated pathways may be integral to effective maternal/fetal functioning during implantation.

We then employed STRING to infer interactions between factors of embryonic and endometrial origin involved in the reported biological processes (Jensen et al., 2009). Focusing on the rba-YMA biological processes, the negative regulation of apoptosis revealed among others, interactions between endometrial EGFR and embryonic UBB, KRT18, and WNT7A, as well as endometrial SOD2 and embryonic PRDX5 (Figure S4; Table S6). Crosstalk between EGF/EGFR and WNT pathways has been reported, consistent with a role in cell migration (Xie et al., 2020; Liu et al., 2017). Apart from their participation in the negative regulation of apoptosis, EGFR is a potential regulator of KRT18 that could also affect cell migration and invasion (Ruan et al., 2011; De Luca et al., 2014; Zhang et al., 2019). Embryonic HSP90AA1 is involved in the immune system pathway (Reactome) (Figure S4) and in signal transduction along with other endometrial factors. Embryonic HSP90AA1 also appears to interact with the endometrial MET, STAT3, LYN, EGFR, RIPK3, ERBB2, ERBB3, and PTGES3 (Figure S4; Table S6). These factors are also involved in cell communication and response to stimulus, with PI3K-Akt signaling (KEGG) pathway including most of the aforementioned endometrial factors along with the embryonic HSP90AA1. This is consistent with increased cell survival in the context of an antiapoptotic environment (Giulino-Roth et al., 2017; Chatterjee et al., 2013).

Following on from the analysis of the most ‘fertile’ system (rba-YMA/receptive endometrium) with a higher potential for implantation success, in order to broaden our understanding on the factors that may limit implantation success, the interactions between rba-AMA and receptive endometria were investigated. Ontological analysis was initially performed on the receptive endometrial transcripts and then on both embryonic rba-AMA and receptive endometrial transcripts together, in the context of a receptive endometrium. The additional biological processes reported when the rba-AMA transcripts are combined with the receptive endometrial transcripts, include among others the *intrinsic apoptotic signaling pathway in response to endoplasmic reticulum stress* (FDR 2.6E-2) and the *keratan sulfate biosynthetic process* (FDR 4.2E-2), and the cellular component *integral component of lumenal side of endoplasmic reticulum membrane* (FDR 4.4E-2) (Table S7). The *apoptotic signaling in response to endoplasmic reticulum stress* could indicate an underlying apoptotic process leading to lower implantation rates compared with the YMA blastocysts, when the AMA blastocysts are transferred into the female uterus (Lin et al., 2019b). The *keratan sulfate biosynthetic process* in the AMA blastocysts may also play a limiting role in embryo implantation, as keratan sulfate is considered an anti-adhesive molecule (Greenwood and Murphy-Ullrich, 1998). Keratan sulfate may affect endothelial cell migration and potentially plays a role during implantation (Funderburgh, 2000). This approach could improve our understanding of the biological processes associated with the AMA/rba-AMA blastocysts undergoing ART with subsequently poor implantation rates.

### 6. Highlighting maternal age features

The DaMiRseq R package for computational analysis (Chiesa et al., 2018) was employed to further refine and highlight informative molecular biomarkers for the selection of the most competent blastocysts with higher implantation success rate. Following gene expression normalization, filtering, and identification of surrogate variables to minimize unwanted variation, a heatmap was generated using Spearman’s correlation, along with an unsupervised multidimensional scaling (MDS) plot, to illustrate gene expression relationships among the three reproductive biological maternal age cohorts (Figures S5A and S5B). Focusing on the two extreme reproductive biological age cohorts, this methodology revealed 290 gene transcripts with distinct expression patterns that could characterize samples belonging to rba-YMA or rba-AMA cohort (FSelect function). Further reduction of highly correlated genes resulted in 58 transcripts that consist a subset of molecular predictors for sample classification using the DaMiRseq FReduct function (Figures S5C and S5D; Table S8). Using only the most informative gene transcripts/predictors to define the relationships among the rba-YMA and rba-AMA cohorts, an MDS plot was generated along with a heatmap relying on the gene expression profiles and illustrating the classification outcome (Figures 3B–3D). Similar to the above ABCG2, DHCR7, SQLE, and ARHGAP10 were among the molecular predictors that could be used to classify rba-YMA and rba-AMA cohorts with greater confidence.

## Discussion

During the last 40 years, the number of AMA women undergoing ART procedures has increased almost four-fold (Lean et al., 2017; Office for National Statistics, 2018). Diagnostic methods employed in ART aimed at improving treatment success rates include mainly preimplantation genetic testing (PGT-A), for the determination of the ploidy status of the blastocysts to be transferred, and morphological criteria, without considering additional factors of potentially great importance. Previous studies have reported a reduction in implantation and delivery rates of euploid blastocysts that were derived from aged women (Scott et al., 2012; Harton et al., 2013). Other studies suggested that chromosomal aneuploidies and blastocyst developmental stage are the main factors affecting implantation rates (Irani et al., 2019). With the proportion of day-5 blastocyst trisomies rising from ∼10% incidence at the age of 30 to ∼20% at the age of 40 (Gruhn et al., 2019), one would expect that the morphologically good quality blastocysts used in the current study had lower rates of aneuploidy (Irani et al., 2019). With 4 out of 36 blastocysts being excluded from the current study due to suspected aneuploidies, our study lies within the observed frequency of aneuploidy.

The blastocyst transcriptome exhibits a maternal chronological age component (Frenk and Houseley, 2018; Jiang et al., 2001; Kawai et al., 2018), even when the embryos are euploid (McCallie et al., 2019). Understanding the underlying causes of the higher incidence of implantation failure among AMA women is integral to reducing the number of women who experience an unsuccessful pregnancy. Comparative human trophectoderm transcriptome profiles were developed as a function of maternal age. Focusing mainly on the young trophectoderm and the receptive endometrial RNA-Seq data, which we combined in the context of a computational model that approximates the more ‘fertile’ human phenotype, we uncovered a corresponding biological age component. These molecular biomarkers could support the development of a diagnostic test for the selection of the most competent blastocysts for embryo transfer. All blastocysts were derived from oocytes fertilized by ICSI. This was intentionally aimed at preserving uniform conditions for all the analysed samples, including cumulus cells dissection. This approach also eliminated potential fluctuations in gene expression levels arising from mixing IVF and ICSI treatments.

Differential gene expression of samples clustered by reproductive biological age, revealed 655 significantly higher expressed gene transcripts in the rba-YMA cohort, with ontologies that could play important roles in implantation. These include the *reduction of oxidative species*, *mitochondrial ATP synthesis*, and *cholesterol biosynthesis*, along with a highly enriched representation of genes involved in *extracellular exosome* biogenesis and function. For example, at the blastocyst stage, mitochondria form an elongated and more active structure reflecting increased cellular metabolism (Sathananthan and Trounson, 2000; Houghton and Leese, 2004), with approximately 80% of the ATP being generated by the trophectoderm (Houghton, 2006). Mitochondrial function/activity has a positive impact on early embryo development (Benkhalifa et al., 2014) and like excessive oxidation mitochondrial dysfunction can compromise embryonic development (Dumollard et al., 2007). As revealed in our study, peroxidases, including glutathione peroxidase 4 (GPX4) and peroxiredoxin 2 (PRDX2), are crucial for early embryo development and participate in an anti-oxidative mechanism that protects the embryo from reactive oxygen species (ROS). Reduced PRDX2 expression has been associated with increased first trimester recurrent miscarriages (Wu et al., 2017).

Cholesterol and steroid biosynthesis are important features of maternal-embryo communication during implantation and placentation (Pusateri et al., 1990; Geisert and Yelich, 1996; Zhou et al., 2019). Cell proliferation is promoted by cholesterol biosynthesis in different stem cell types (Wang et al., 2018; Jian-Feng et al., 2012; Ehmsen et al., 2019; Oguro, 2019), consistent with cholesterol and its metabolites serving a functional role in the YMA trophectoderm cell layer. Endometrial receptivity is dependent on steroid hormones (Bagchi et al., 2001) that can impact exosome biogenesis, as they encapsulate various signals prior to their release, distribution and uptake by target cells (Pfrieger and Vitale, 2018). Exosomes, for example, released into the endometrial microenvironment could be derived either from the blastocyst or the endometrium and both may carry signals necessary for embryo-maternal communication (Saadeldin et al., 2015; Desrochers et al., 2016; Homer et al., 2017; Adam et al., 2017).

A large morphogenetic divergence distinguishes mouse from human development at and following implantation, including among others the blastocyst region that initiates implantation i.e. the embryonic (human) and abembryonic (mouse) poles and the primate specific formation of terminally differentiated trophoblast cell types. Despite these differences, there is considerable conservation of gene expression during mouse and human embryo implantation, allowing the use of the mouse model to aid interpretation of the human implantation process with caution (Cha et al., 2012). As confirmed by knockout mouse data (Table S9), several human exosome-related genes revealed in the current study have potentially important roles in early embryo development and implantation success. Gene transcripts represented in these ontologies could present a set of predictive molecular biomarkers of blastocysts with higher implantation potential. These molecular processes involved 351 rba-YMA trophectoderm gene transcripts (or 106 genes), most of which interact with the receptive endometrial factors that could be used to predict with higher confidence the blastocysts with higher likelihood to implant. Confidence in this prediction increases when using only the 120 transcripts (35 genes) identified in both analyses detailed above. The gene transcripts significantly more highly expressed in the rba-AMA cohort could therefore be considered as indicators of lower implantation success rate, along with the *intrinsic apoptotic signaling pathway* and *keratan sulfate biosynthetic process*.

Transcriptomes derived from the receptive endometrium and YMA/rba-YMA trophectoderm were enriched in genes that regulate apoptosis. This emphasizes the need for a fine-tuned balance between pro- and anti-apoptotic signals, for implantation into the endometrial microenvironment. For example, uterine epithelial cell apoptosis is subject to autocrine/paracrine regulation (Kamijo et al., 1998) that in turn can be influenced by the levels of genes regulating apoptosis in the AMA/rba-AMA trophectoderm. Perturbation of apoptosis can lead to implantation failure. This is typified by the anti-apoptotic *Mcl1* gene knockout mouse model that appears deficient for a supportive uterine environment (Rinkenberger et al., 2000). This finding is consistent with YMA/rba-YMA and receptive endometrial factors collaboratively regulating apoptosis. Interestingly, from an embryonic perspective, the regulation of apoptosis suggests that younger, implantation competent blastocysts together with endometrial factors suppress trophectoderm apoptosis and increase blastocyst stability. Other mediators regulating the implantation pathway include the embryonically expressed genes *DAB2, CD274*, and *HSP90AA1* that together with specific maternal genes participate in *signal transduction* and *positive regulation of cell migration*. It is well known that endometrial cell migration plays an important role in embryonic implantation (Grewal et al., 2008). Together with our findings, this supports the suggestion that apart from early embryonic signal transduction, Day 5 trophectoderm could predetermine and induce receptive endometrial cell migration, potentially facilitating human embryo implantation. Among ∼170 proteins reported in the blastocoele following blastocentesis (Poli et al., 2015), 8 rba-YMA factors including the extracellular exosome-related HSP90AA1, PRDX2, PSMA5, TAGLN2, FABP5, and PSMA7 were also found in our data. This provides linkage between our study (RNA level) and the study by Poli et al. (protein level) supporting the likelihood of embryo-endometrial interactions involving the extracellular space.

The current study is the closest high-throughput approach for examining embryo-endometrial interactions without requiring any unethical, *in vivo* experimentation on humans. Our experimental design provides insights into the impact of aging on the dynamics of the trophectoderm transcriptome, revealing significantly higher implantation success of the rba-YMA cohort (two-tailed Fisher’s test, p=0.012). Potential mechanisms involved in the non-conserved (Bischof et al., 2000; Dey et al., 2004) implantation process, were born out in the context of a receptive endometrium. This resolved as exosome-related factors that probably promote cell adhesion and migration within an anti-apoptotic environment of efficient energy production, when a steroid biosynthetic mechanism is in place. The novel biological processes and overall insights into the YMA/rba-YMA trophectoderm and receptive endometrial factors uncovered potential molecular biomarkers of implantation success and/or early embryo development competency. The results of the current study powered (70%) provide the basis for extension to a larger prospective analysis. Our molecular biomarkers could be further tested on a clinical basis through the simultaneous examination of the genome (PGT-A) and transcriptome at biopsy, permitting the full potential of molecular methods in identifying the most competent blastocysts for embryo transfer. In conclusion, the biomarkers revealed in this study could form the basis of a clinical diagnostic test to select the most competent blastocysts for uterine transfer with a higher probability of successful implantation and subsequent development.

### Limitations of the study

Embryo implantation is a complex process that cannot be directly studied in humans, while it can be examined *in vitro*. In the context of a receptive endometrium and maternal age, we examined whether blastocyst implantation success reflects the trophectoderm transcriptome. We attempted to mimic the *in vivo* environment and appreciate that reconstructing this complex process is not entirely complete. Accordingly, we coupled a high-throughput *in vitro* approach with computational analyses to examine embryo-endometrial communication during implantation, as a function of maternal age. Within this framework several factors were revealed that could play a role in embryo-endometrial interactions. IVF patients are understandably hesitant when consenting for research purposes, hence, clinical samples were relatively scarce, limiting the study’s scope. Increasing the sample size may have revealed additional factors that play a role in embryo-endometrial communication, embryonic development, and implantation success and/or strengthened those reported herein.

## STAR★Methods

### Key resources table

**Table:** 

REAGENT or RESOURCE	SOURCE	IDENTIFIER
**Chemicals, peptides, and recombinant proteins**		

Continuous single culture media	IrvineScientific	90164
Human Serum Albumin (HSA) solution	IrvineScientific	9988
Agencourt Ampure XP Beads	Beckman Coulter	A63880
Nextera XT Index Kit (24 indexes, 96 samples)	Illumina	FC-131-1001

**Critical commercial assays**		

Human Chorionic Gonadotropin (hCG) assay	Roche Diagnostics International	21198-7
SMART-Seq v4 Ultra Low Input RNA assay	Takara-Clontech	634890
Qubit dsDNA high sensitivity fluorometric assay	Thermo Fisher Scientific	Q32851
Nextera XT DNA library preparation assay	Illumina	FC-131-1024
Bioanalyzer High Sensitivity DNA assay	Agilent	5067-4626
SYBR Green MasterMix	Thermo Fisher Scientific	13256519

**Deposited data**		

Raw and process data	This paper	GEO: GSE133592

**Oligonucleotides**		

Primers for RT-qPCR, see Table S10	This paper	N/A

**Software and algorithms**		

FastQC	Andrews, 2010	https://www.bioinformatics.babraham.ac.uk/projects/fastqc/
Trim galore	Krueger, 2015	https://www.bioinformatics.babraham.ac.uk/projects/trim_galore/
HISAT2	Pertea et al., 2016	http://daehwankimlab.github.io/hisat2/
Samtools	Li et al., 2009	http://samtools.sourceforge.net/
Picard tools	Broad Institute, 2010	ttp://broadinstitute.github.io/picard
StringTie	Pertea et al., 2015	https://ccb.jhu.edu/software/stringtie/
Python script to extract read counts from the HISAT2/StringTie outputs in a format suitable as input for the Bioconductor R package edgeR	Pertea et al., 2015	https://ccb.jhu.edu/software/stringtie/dl/prepDE.py
edgeR	(Robinson and Oshlack, 2010	https://bioconductor.org/packages/release/bioc/html/edgeR.html
featureCounts	Liao et al., 2013	https://www.rdocumentation.org/packages/Rsubread/versions/1.22.2/topics/featureCounts
Clustvis	Metsalu and Vilo, 2015	https://biit.cs.ut.ee/clustvis/
Plotly	Plotly Technologies Inc	https://plotly.com/
heatmap.plus	Day, 2012	https://www.rdocumentation.org/packages/heatmap.plus/versions/1.3/topics/heatmap.plus.package
DaMiRseq	Chiesa et al., 2018	https://bioconductor.org/packages/release/bioc/html/DaMiRseq.html
ggplot2	Wickham, 2016	https://ggplot2.tidyverse.org/
Pheatmap	Kolde and Kolde, 2019	https://www.rdocumentation.org/packages/pheatmap/versions/1.0.12/topics/pheatmap
Cytoscape GeneMANIA	Mostafavi et al., 2008, Warde-Farley et al., 2010	http://apps.cytoscape.org/apps/genemania
DAVID	Huang et al., 2009a, Huang et al., 2009b	https://david.ncifcrf.gov/
STRING	Jensen et al., 2009	https://string-db.org/
RnaSeqSampleSize	Zhao et al., 2018	http://bioconductor.org/packages/release/bioc/html/RnaSeqSampleSize.html
eSNP karyotyping protocol	Weissbein et al., 2016, Ntostis et al., 2019	https://github.com/daveiles/human_TEbiopsy_eSNPanalysis
GATK	McKenna et al., 2010	https://gatk.broadinstitute.org/hc/en-us

### Resource availability

#### Lead contact

Further information and requests for resources and reagents should be directed to and will be fulfilled by the lead contact, Panagiotis Ntostis (P.Ntostis@leeds.ac.uk).

#### Materials availability

This study did not generate new unique reagents.

#### Data and code availability

The RNA sequencing data sets generated during this study are available at the NCBI Gene Expression Omnibus (GEO) repository under the accession number GSE133592 (https://www.ncbi.nlm.nih.gov/geo/). The current study did not use any new code.

### Experimental model and subject details

Trophectoderm cells biopsied from day 5 blastocysts were donated with informed consent sought from couples attending for infertility treatment. Ethical approvals were issued by the National Health System, A’ Administration of the Health District of Attica, Greece, General Children Hospital ‘Aghia Sofia’ (Reference – Protocol Number: 19964/04-09-2014), the Greek National Authority of Assisted Reproduction and the bioethics committee of Genesis Athens clinic. Additional information related to the subjects can be found in the results section ‘Clinical samples’. All samples were recruited from a single reproductive medicine unit (Genesis Athens Clinic, Greece).

### Method details

#### Εmbryo culture, blastocyst biopsy procedure and clinical outcome

Oocytes were fertilized by intracytoplasmic sperm injection (ICSI) and cultured in continuous single culture media with 5% human serum albumin (HSA) (Irvine Scientific, CA, USA) to the blastocyst stage. Blastocysts were evaluated according to their degree of expansion and the quality of their inner cell mass (ICM) and trophectoderm with 3-5AA considered as excellent, 3-5B∗ as good and 3-5BB as average quality while C∗ was considered poor quality (Balaban et al., 2011; Gardner and Schoolcraft, 1999). Our study employed average embryo quality as the minimum morphological grade used in all cohorts (Gardner and Schoolcraft, 1999).

Trophectoderm biopsy was performed with each blastocyst positioned on the holding pipette and oriented in a way that the ICM was clearly visible and on the side opposite the biopsy pipette (Cook Medical, Bloomington, USA) (Kokkali et al., 2007). Trophectoderm cells from young maternal age (YMA, <30 yo), intermediate maternal age (IMA, 30-39 yo) and advanced maternal age (AMA, >40 yo) blastocyst cohorts were gently aspirated into the biopsy pipette with moderate suction, before immediate transfer into the supplier’s lysis buffer (10X) containing RNase Inhibitor (Takara-Clontech, USA) and freezing at −80°C until required.

Embryo implantation of the transferred blastocysts into the female endometrium was determined by serum beta hCG levels (≥25 mIU/mL) (Roche Diagnostics International, Switzerland) on Day 16 after oocyte retrieval and confirmed by demonstration of a gestational sac by ultrasound scan, performed 4 weeks after embryo transfer. Clinical pregnancy was defined as the presence of a fetal heartbeat beyond 7 weeks of gestation.

#### RNA isolation, cDNA library construction and sequence processing

Library construction used the SMART-Seq v4 Ultra Low Input RNA method (Takara-Clontech, USA) following the manufacturer’s instructions. First strand cDNA was synthesized using the SMART-Seq locked nucleic acid (LNA) technology and the full-length cDNA amplified by long-distance PCR (LD-PCR). To permit accurate comparisons among samples and minimize PCR bias, an optimized constant number of 13 PCR cycles was applied throughout using the same amount of RNA in each reaction (Ntostis et al., 2019). The amplified cDNA was then purified using Agencourt Ampure XP Beads (Beckman Coulter, USA).

Qubit dsDNA high sensitivity fluorometric assay was employed to quantify the amplified cDNA (Thermo Fisher Scientific, USA). Approximately 150 pg of cDNA were processed using Illumina’s Nextera XT DNA library preparation method, according to the manufacturer’s instructions (Illumina, USA). A ‘tagmentation’ step (tag and DNA fragmentation in a single step) was then applied to the full-length cDNA products allowing unique index adapter combinations to be generated for each library. The average fragmented cDNA library length distribution was assessed using the high sensitivity DNA assay protocol (Agilent, USA). Following equimolar pooling, paired-end deep sequencing was employed on a Hi-Seq 3000 instrument (Illumina, USA), generating 150 bp reads.

Sequence quality was assessed by FastQC (Andrews, 2010) and Trim Galore (Krueger, 2015) was employed for automated Nextera adapter and quality trimming. Reads passing quality control (QC) were mapped to the human reference genome (hg38) using the HISAT2 aligner v2.1.0 (Pertea et al., 2016). Samtools v1.8 was employed to remove unmapped and unpaired reads (Li et al., 2009) and PCR duplicates were tagged by Picard tools v2.1.1 Broad Institute.(2010). Available online at http://broadinstitute.github.io/picard. StringTie was used to detect potentially novel transcripts, using an hg38 annotation file (UCSC) of the human transcriptome as a guide (Pertea et al., 2015). To cross-check the robustness of DE analysis, two approaches were employed to assign and quantify read mapping to known and predicted genes. The first used a python script (PythonScript, 2018) to extract read counts from the HISAT2/StringTie outputs in a format suitable as input for the Bioconductor R package edgeR v3.9 (Robinson and Oshlack, 2010). The other used the featureCounts function of Rsubread to generate count tables (Liao et al., 2013). Only uniquely mapped, correctly paired and non-duplicated reads were considered.

#### Bioinformatics analysis, gene networks, molecular interactions and functional annotation analysis

Using Clustvis (Metsalu and Vilo, 2015, Available online at https://biit.cs.ut.ee/clustvis/), the transcriptome of YMA (<30 yo), IMA (30–39 yo) and AMA (>40 yo) were clustered using PCA. Briefly, Pareto scaling was performed using the singular value decomposition with imputation method for principal component (PC) calculation. PC1 and PC2 alone explained 55.3% of the variance between samples. For visualization of the data in 3 dimensions; PC1, PC2 and PC3 were extracted and visualized using the plot_ly() function in R (Plotly Technologies Inc, 2015). Ηeatmap was constructed based on the chronological maternal age; YMA, IMA, and AMA, to depict the differential gene expression patterns among the three cohorts, using the heatmap.plus R package (Day, 2012). The reproductive biological age (rba); rba-YMA, rba-IMA and rba-AMA, was determined by unsupervised hierarchical clustering analysis of the trophectoderm gene expression. Hierarchical clustering was generated from the logarithm fold-change (logFC) data for the differentially expressed genes among rba-YMA, rba-IMA, and rba-AMA cohorts. The samples comprising the YMA/rba-YMA and AMA/rba-AMA cohorts, along with the implantation success rates between rba-YMA and rba-AMA, supported the reproductive biological age classification.

The DaMiRseq R package pipeline for computational analysis was employed to determine molecular predictors that could characterize samples of reproductive young and advanced maternal age (Chiesa et al., 2018). We used a correlation cutoff of 0.3 for normalization and partial least-square feature selection (FSelect function) to consider gene expression in at least half of the samples of the smaller (rba-AMA) cohort. We selected the number of surrogate variables that explained at least 90% of the variation. Default values were used for other functions. R packages were employed for generating distance matrix and multi-dimensional scaling plots (Wickham, 2016; Kolde and Kolde, 2019).

A methodological approach that could reveal the potential relationship between the trophectoderm and endometrial transcriptomes was designed (Figure 4A). Ontological analysis of the significantly differentially expressed genes between YMA, IMA, and AMA cohorts were explored using DAVID (Huang et al., 2009a, 2009b) for enriched cellular components and biological processes. False discovery rate (FDR) was used for multiple comparison corrections in all analyses. Attention was given to those significant ontologies most likely describing maternal-fetal communication. The top significant ontologies from the YMA trophectoderm cohort in conjunction with the significantly more highly represented ontologies (cellular component) of the corresponding receptive endometria (luteinizing hormone LH+7 or LH+8) were investigated further (Figure 4A). The enriched extracellular exosome genes were verified using ExoCarta (Keerthikumar et al., 2016) and EBI (www.ebi.ac.uk/QuickGO/GProteinSet?id=Exosome, Accessed on May 2019) databases.

Gene expression products participating in the aforementioned ontologies were assessed with Cytoscape GeneMANIA v3.4.1 module (Mostafavi et al., 2008; Warde-Farley et al., 2010). This software uses publicly available datasets representing different molecular interactions, molecular pathways and other data types. To refine maternal-fetal communication pathways, only trophectoderm and endometrial protein-coding factors with known physical interactions and/or sharing common pathways were considered as part of the current methodology. The final stage of the pipeline employed an additional ontological analysis (biological process) of the potentially interacting endometrial and trophectoderm factors using DAVID (Huang et al., 2009a, 2009b). Here, genes were analyzed as a single group to reveal the common significant biological processes that maternal and fetal factors may share when exosomes of fetal origin fuse with endometrial cells and vice versa. STRING (Jensen et al., 2009) was employed to further investigate the interactions among the maternal and fetal factors that participate in common biological processes, providing a better understanding of the potential interactions between exosome- and extracellular-related factors of maternal and fetal origin.

#### Endometrial RNA sequencing meta-analysis

Using RNA sequencing, Hu and colleagues (2014) reported significantly differentially expressed (DE) genes between pre-receptive (LH+2) and receptive endometria (LH+7). Altmae and colleagues (2017) also reported DE genes between pre-receptive (LH+2) and receptive endometria (LH+8) (Hu et al., 2014; Altmäe et al., 2017). RNA-Seq data from Altmae study was downloaded from the public database Gene Expression Omnibus (GEO) (www.ncbi.nlm.nih.gov/geo/), under the accession number GSE98386. The current study used this data in the context of meta-analysis, focusing on the significantly highly expressed genes in the receptive endometria and revealing their potential interactions with the young trophectoderm factors.

### Quantification and statistical analysis

The edgeR *calcNormFactors* function was used to normalize count data, based on the trimmed mean M value (TMM) (Robinson and Oshlack, 2010). The thresholds for gene expression inclusion in the DE analysis were at least 5 (or 1) counts-per-million (cpm) reads present in at least 4 samples. Genes expressed at significantly different levels between YMA/IMA and AMA trophectoderm cohorts, as well as receptive and non-receptive endometria were reported using edgeR’s generalized linear model (GLM) for the trophectoderm, or exact test for the endometrium with the FDR set at 0.05 (Robinson and Oshlack, 2010). The mean paternal age was compared among the 3 age cohorts using ANOVA in R (aov() function). Blastocyst developmental stage was compared among the 3 maternal age cohorts, using Kruskal Wallis test (kruskal.test() function) in R Stats package (Team, 2019). Normality of age and blastocyst grade variables were assessed using Shapiro-Wilks test (Stats package). Fisher’s exact test was employed to investigate the association between trophectoderm gene expression based on the maternal age and implantation success (Stats package). RnaSeqSampleSize package was used to calculate the power of the 32 samples recruited by the study (Zhao et al., 2018).

#### Aneuploidy detection analysis by RNA sequencing data

We used a previously described workflow (Weissbein et al., 2016), with some modifications (Ntostis et al., 2019), to determine the ploidy status of the human blastocysts recruited by the current study. The scripts and complete workflow are available at https://github.com/daveiles/human_TEbiopsy_eSNPanalysis. Briefly, correctly paired, mapped and oriented HISAT2 alignments (http://daehwankimlab.github.io/hisat2/about/) were processed to generate single nucleotide variant calls in vcf format from mapped reads as follows. PCR duplicates were detected and removed with Picard MarkDuplicates and read group information was added with Picard AddOrReplaceReadGroups (both tools accessible at https://broadinstitute.github.io/picard/). The GATK v4.1.0.0 tools SplitNCigarReads and HaplotypeCaller (McKenna et al., 2010), were used to create vcf files for downstream analyses to detect chromosomal anomalies and loss of heterozygosity (LOH) as described by Weissbein and colleagues, using snp151Common (downloaded from the UCSC Table Browser) as a reference. Reported varied allelic ratios during the aneuploidy analysis around peri-centromeric regions, could be explained by low levels of mosaicism, lower expression levels of peri-centromerically located genes and/or the low initial RNA inputs and together with viable trisomies were retained in the analysis.

#### RNA sequencing validation by RT-qPCR

RNA sequencing validation involved reverse transcription of the biopsied trophectoderm RNA using the SMART ultra-low input RNA protocol (Takara-Clontech, USA). The cDNA was amplified by long distance PCR (LD-PCR) generating sufficient quantities for the RNA sequencing validation. Reverse transcription quantitative PCR (RT-qPCR) was conducted using the thermal cycler LC480 (Roche, Switzerland). Three biological replicates were used per cohort. The annealing temperature per primer pair was set at 60°C (Table S10). Gene expression data generated by RT-qPCR were normalized against the average gene expression levels of the endogenous controls (housekeeping genes) *ACTB*, *GAPDH*, *H3F3B*, and *HNRNPC.* Fold-change between the YMA/rba-YMA and AMA/rba-AMA samples was calculated using the 2ˆ-ΔΔCt method (Livak and Schmittgen, 2001).

## Supporting citations

Borchert et al. (2006); Dickinson et al., 2016; Imai and Nakagawa (2003); Morris et al. (2002); Turan and Bastepe (2013); Mu et al. (2010); Yant et al., 2003.

## Supplemental information

Document S1. Figures S1–S5

Table S1. Comparison between YMA vs AMA and IMA vs AMA differentially expressed (DE) protein-coding transcripts identified using a generalized linear model (GLM),related to Figure 2

Table S2. Differentially expressed trophectoderm transcripts between YMA and AMA groups,related to Figures 2 and 3 (A) Transcripts significantly higher expressed in the YMA group. (B) Transcripts significantly higher expressed in the AMA group

Table S3. Differentially expressed gene transcripts between young reproductive biological maternal age (rba-YMA) and advanced reproductive biological maternal age (rba-AMA) group,related to Figures 2 and 3 (A) Transcripts significantly higher expressed in the rba-YMA group. (B) Transcripts significantly higher expressed in the rba-AMA group.

Table S4. Biological process and cellular component ontologies of the significantly higher gene transcripts in young maternal age (YMA), advanced maternal age (AMA) cohort, young maternal biological age (rba-AMA) and young maternal biological age (rba-AMA) were reported,related to Figures 2, 3, and 4

Table S5. Gene product interactions identified by GeneMANIA Cytoscape module between maternal (M) and fetal (F) products of chronological and reproductive biological maternal age,related to Figure 4 and STAR methods

Table S6. Significant biological processes (BP) involving the maternal (receptive endometrium) and fetal (young maternal age – YMA or young biological maternal age – rba-YMA) gene products with determined physical interactions according to Cytoscape GeneMANIA module,related to Figure 4 and STAR Methods The maternal (M) or fetal (F) origin is reported per gene product, along with the number of genes per ontological term (count), the P-Value, Benjamini and false discovery rate (FDR) reported by DAVID.

Table S7. Gene ontologies that include fetal (young biological maternal age – rba-YMA), maternal (receptive endometrium) and embryo-endometrial genes (receptive and rba-AMA) with corresponding biological processes (BP) and cellular components (CC) according to DAVID,related to Figure 4 and STAR Methods

Table S8. Subset of transcripts derived from DaMiRseq R package, that could be used to separate trophectoderm samples by maternal age,related to Figure 4 and STAR Methods RefSeq ID and associated gene names are displayed persubset of 290 (FSelect function) and 58 (FReduct function) transcripts.

Table S9. Report of trophectoderm genes significantly more highly expressed in young maternal age (YMA) women, along with corresponding knockout mouse studies,related to Figure 4

Table S10. DNA oligo sequences (primers) used to validate the RNA sequencing results by RT-qPCR,related to Figure 2 and STAR Methods Gene names arereported together with the forward/reverse primer sequences. ACTB, GAPDH, H3F3Band HNRNPC were used as reference genes (endogenous controls).
