# Supplementary material for: The effects of aging on molecular modulators of human embryo implantation
Source: iScience. 2021 Jun 19;24(7):102751. doi: 10.1016/j.isci.2021.102751 (PMC8271113; doi:10.1016/j.isci.2021.102751)
Supplement: Document S1. Figures S1–S5 [file mmc1.pdf]

iScience, Volume *24*

Supplemental information

The effects of aging on molecular modulators of human embryo implantation

Panagiotis Ntostis, Grace Swanson, Georgia Kokkali, David Iles, John Huntriss, Agni Pantou, Maria Tzetis, Konstantinos Pantos, Helen M. Picton, Stephen A. Krawetz, and David Miller

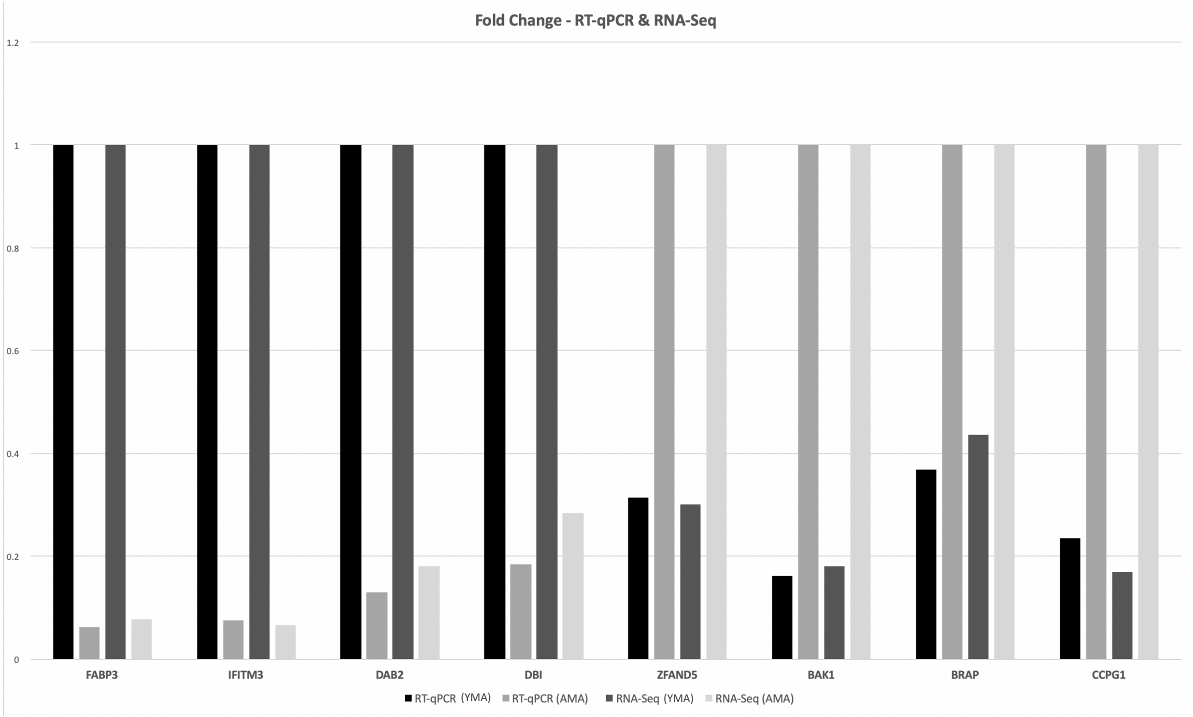

Figure S1. Histogram illustrating the fold-change levels in YMA samples using RT-qPCR (black) and RNA-Seq (dark grey) and in advanced maternal age (AMA) samples by RT-qPCR (grey) and RNA-Seq (light grey) methods. Related to Figure 2 and STAR methods.

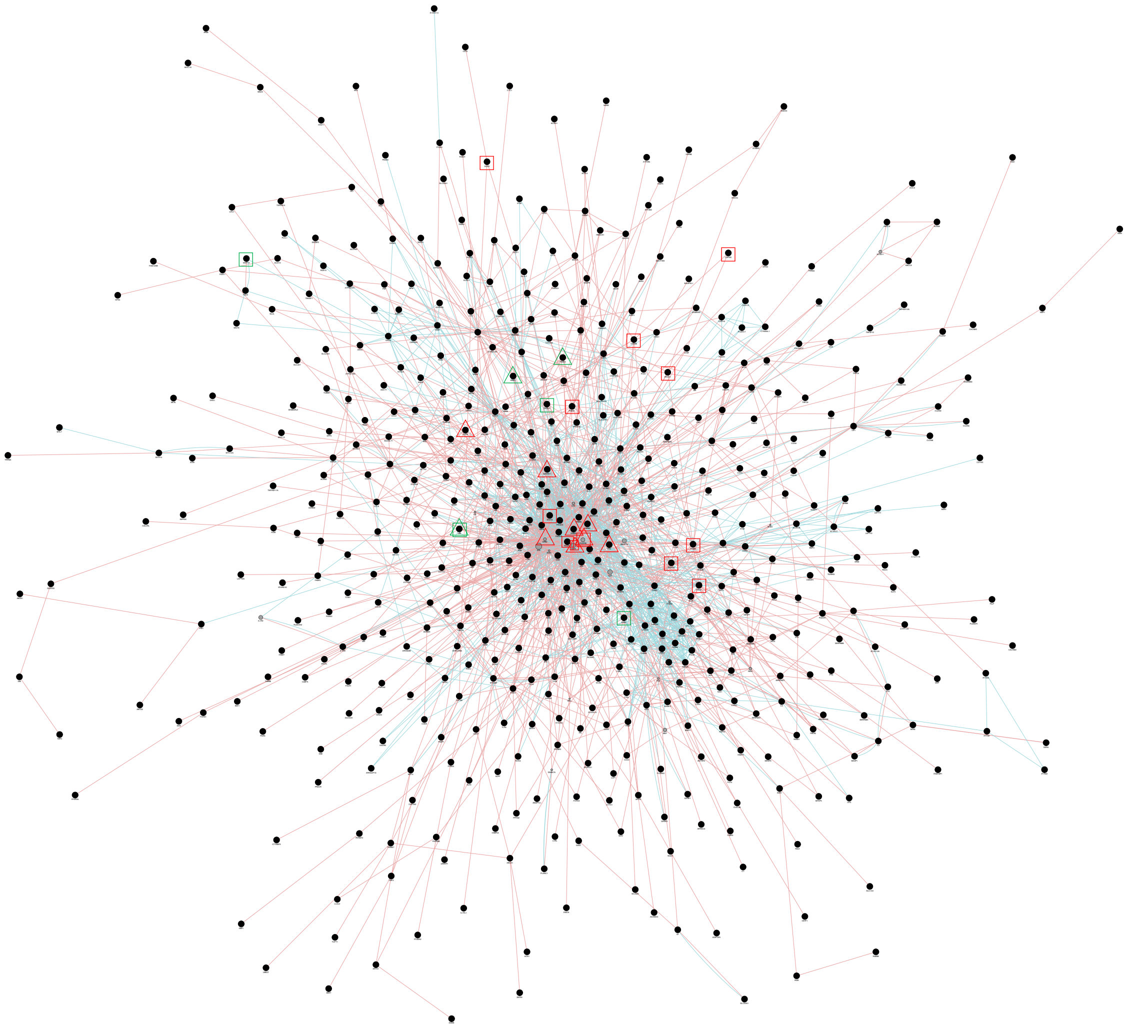

Figure S2. Full interconnected network of the trophectoderm YMA samples and the receptive endometrium exosome- and plasma membrane-related genes. Related to Figure 4 and STAR methods. GeneMANIA analysis was applied to the interconnected network of the exosome-related genes that were more highly expressed in the trophectoderm of YMA women and in the receptive endometrium. The gene products highlighted indicate maternal (red) and embryonic (green) origin. Regulation of apoptosis (squares) and MAPK cascade (triangles) ontologies are displayed. Black nodes represent the identified exosome- and plasma membrane-related genes. Grey nodes depict transcripts with underlying interactions with the

black nodes. Potential interactions are displayed by interconnecting lines. Interconnected line colours signify the interaction type including physical interactions (red) and common biological pathways (blue).

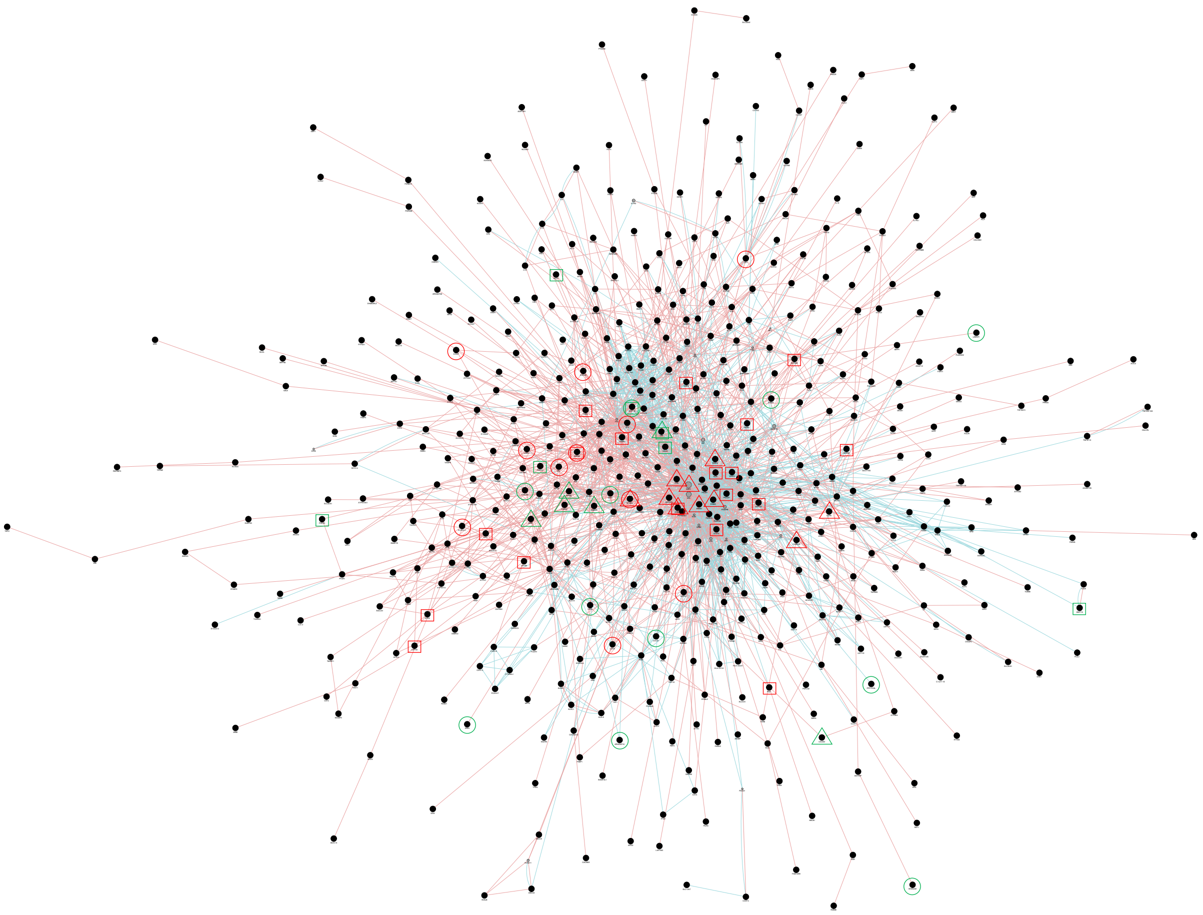

Figure S3. Full interconnected network of the trophectoderm rba-YMA and receptive endometrium exosome- and plasma membrane-related genes. Related to Figure 4 and STAR methods. Interconnected network of the young maternal reproductive biological age (rba-YMA) and receptive endometrium exosome- and plasma membrane-related genes. The gene products highlighted

indicate maternal (red) or embryonic (green) origin. Cell-cell adhesion (circles), regulation of apoptosis (squares) and MAPK cascade (triangles) ontologies are displayed. Black nodes represent the identified exosome- and plasma membrane-related genes. Grey nodes depict transcripts with underlying interactions with the black nodes. Potential interactions are displayed by interconnecting lines. Interconnected line colours signify the interaction type including physical interactions (red) and common biological pathways (blue).

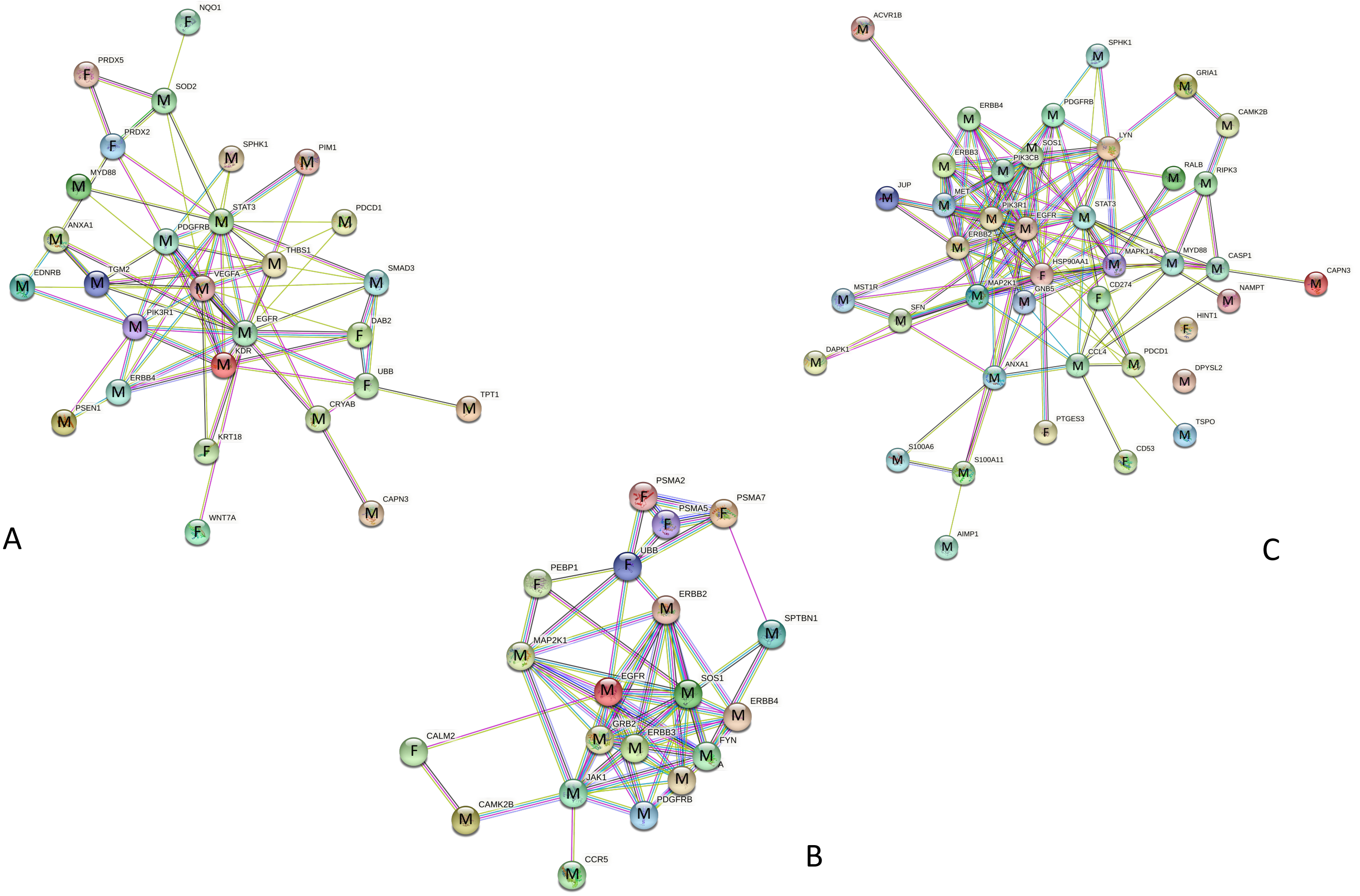

Figure S4. STRING networks illustrating the interactions between the maternal/endometrial (M) and fetal/embryonic (F) factors. Related to Figure 4

and STAR methods. A. Network illustrating maternal and fetal factors involved in the negative regulation of apoptotic process. B. Network illustrating maternal and fetal factors involved in the MAPK cascade. C. Network illustrating maternal and fetal factors involved in the biological process of signal transduction. The origin of each factor is characterised by the letter M (Maternal) and F (Fetal). The edges represent known interactions from curated databases (light blue) or experimentally determined (pink), predicted interactions relied on gene neighbourhood (green), gene fusions (red) and gene co-occurrence (blue) and others, including text mining (light green), co-expression (black) and protein homology (purple).

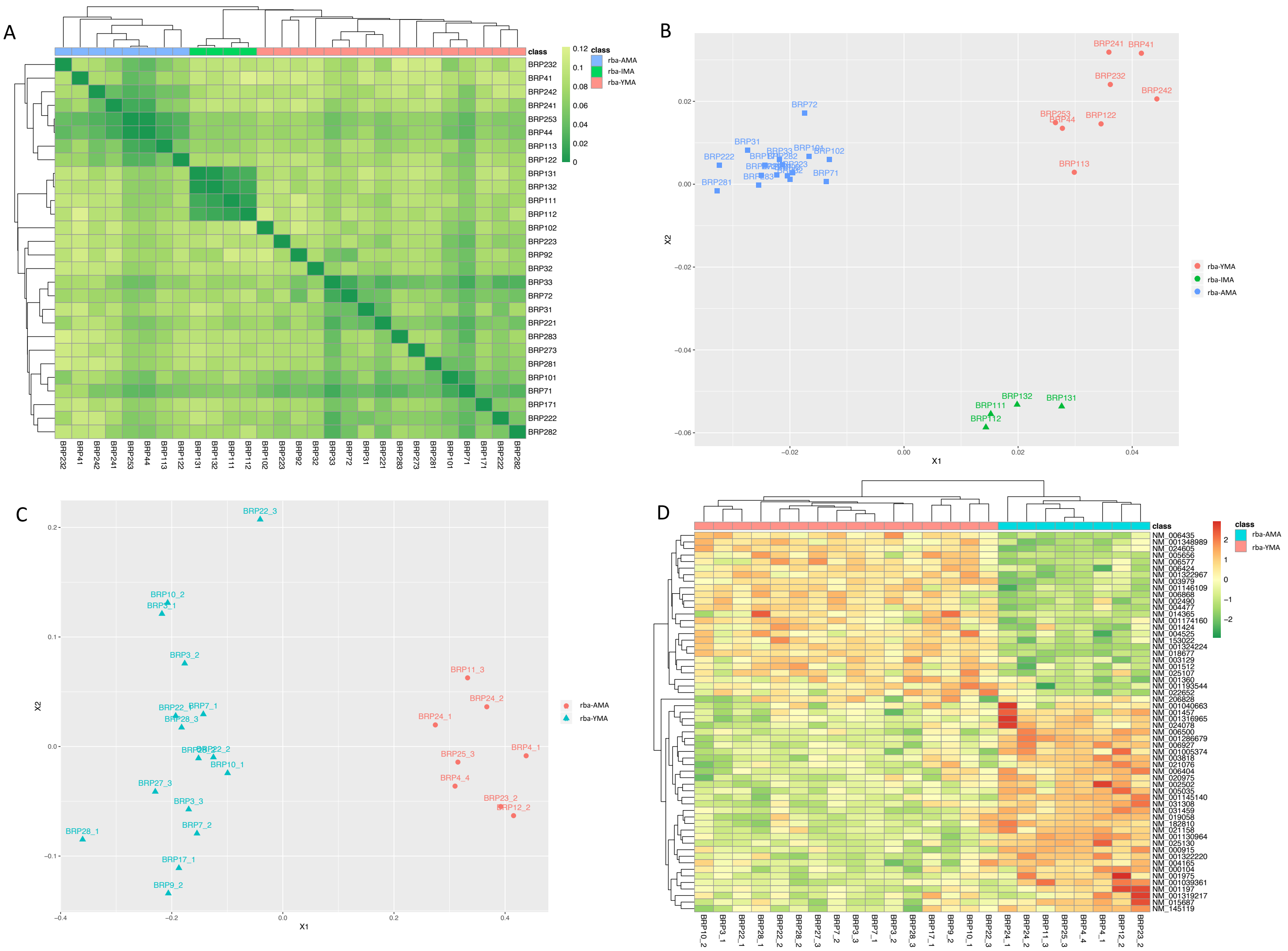

Figure S5. Quality control and feature selection. Related to Figure 4 and STAR methods. A. Heatmap with colours highlighting the distance matrix. Spearman's correlation metric was used. Colour gradient ranges from dark green (minimum) to light green (maximum) distance. Horizontal bars (on the top of heatmap) represent the maternal age class and cluster identification is indicated by the two dendrograms. B. Unsupervised MDS plot illustrating samples coloured according to the reproductive maternal age class. C. A MDS plot of rba-YMA and rba-AMA cohorts, considering only the potentially most informative genes obtained after feature selection. D. Heatmap generated using the expression values of the 58 predictors for rba-YMA and rba-AMA cohorts, revealed by the FReduct function. The

two dendrograms illustrate hierarchical relationships among rba-YMA and rba-AMA cohorts (top) and molecular biomarkers (left). Colour gradient ranges from red (higher expression) to green (lower expression).
