# Supplementary material for: The effects of aging on molecular modulators of human embryo implantation
Source: iScience. 2021 Jun 19;24(7):102751. doi: 10.1016/j.isci.2021.102751 (PMC8271113; doi:10.1016/j.isci.2021.102751)
Supplement: Table S4. Biological process and cellular component ontologies of the significantly higher gene transcripts in young maternal age (YMA), advanced maternal age (AMA) cohort, young maternal biological age (rba-AMA) and young maternal biological age (rba-AMA) were reported,related to Figures 2, 3, and  [file mmc5.pdf]

Table S4. Biological process and cellular component ontologies of the significantly higher gene transcripts in young maternal age (YMA), advanced maternal age (AMA) cohort, young maternal biological age (rba-AMA) and young maternal biological age (rba-AMA) were reported. Related to Figure 2, Figure 3 and Figure 4.

**YMA DE gene transcripts (Chronological age)**			**YMA DE gene transcripts (Chronological age)**		
**Biological Process**			**Cellular Component**		
**Ontological term**	**P-Value**	**FDR**	**Ontological term**	**P-Value**	**FDR**
isoprenoid biosynthetic process	8.2E-5	1.2E-1	extracellular exosome	6.8E-9	8.2E-6
cholesterol biosynthetic process	9.0E-5	1.4E-1	cytosol	2.1E-4	2.6E-1
lipid metabolic process	4.6E-4	6.8E-1	late endosome membrane	3.0E-3	3.5E0
Wnt signaling pathway	1.1E-3	1.7E0	endocytic vesicle membrane	6.9E-3	8.0E0
response to interferon-beta	1.4E-3	2.0E0	ruffle	1.6E-2	1.8E1
**AMA DE gene transcripts (Chronological age)**			**AMA DE gene transcripts (Chronological age)**		
**Biological Process**			**Cellular Component**		
**Ontological term**	**P-Value**	**FDR**	**Ontological term**	**P-Value**	**FDR**
positive regulation of interleukin-6 production	2.2E-3	3.3E0	integral component of Golgi membrane	4.1E-2	3.9E1
regulation of I-kappaB kinase/NF-kappaB signaling	3.3E-3	4.8E0	neurofilament	4.3E-2	4.1E1
endocrine pancreas development	9.8E-3	1.4E1	mitochondrial outer membrane	5.0E-2	4.6E1
**rba-YMA DE gene transcripts (Reproductive biological age)**			**rba-YMA DE gene transcripts (Reproductive biological age)**		
**Biological Process**			**Cellular Component**		
**Ontological Term**	**P-Value**	**FDR**	**Ontological Term**	**P-Value**	**FDR**
cholesterol biosynthetic process	1.9E-15	3.1E-12	extracellular exosome	1.3E-20	1.7E-17

mitochondrial ATP synthesis coupled proton transport	3.0E-7	4.8E-4	cytosol	2.7E-6	3.5E-3
isoprenoid biosynthetic process	8.7E-7	1.4E-3	mitochondrial inner membrane	5.1E-6	6.6E-3
ATP synthesis coupled proton transport	1.0E-5	1.7E-2	mitochondrial proton-transporting ATP synthase complex	6.0E-6	7.8E-3
ATP biosynthetic process	4.4E-5	7.1E-2	mitochondrion	2.0E-5	2.6E-2
lipid metabolic process	6.6E-5	1.1E-1	cell-cell adherens junction	8.6E-5	1.1E-1
cell-cell adhesion	1.1E-4	1.7E-1	mitochondrial matrix	9.8E-5	1.3E-1
**rba-AMA DE gene transcripts (Reproductive biological age)**			**rba-AMA DE gene transcripts (Reproductive biological age)**		
**Biological Process**			**Cellular Component**		
**Ontological Term**	**P-Value**	**FDR**	**Ontological Term**	**P-Value**	**FDR**
autophagosome assembly	5.0E-5	7.9E-2	mitochondrial outer membrane	3.3E-3	4.2E0
macroautophagy	1.1E-4	1.7E-1	pre-autophagosomal structure membrane	1.0E-2	1.2E1
intrinsic apoptotic signaling pathway in response to endoplasmic reticulum stress	3.0E-4	4.8E-1	pre-autophagosomal structure	1.4E-2	1.7E1
