# Supplementary material for: The effects of aging on molecular modulators of human embryo implantation
Source: iScience. 2021 Jun 19;24(7):102751. doi: 10.1016/j.isci.2021.102751 (PMC8271113; doi:10.1016/j.isci.2021.102751)
Supplement: Table S8. Subset of transcripts derived from DaMiRseq R package, that could be used to separate trophectoderm samples by maternal age,related to Figure 4 and STAR Methods — RefSeq ID and associated gene names are displayed persubset of 290 (FSelect function) and 58 (FReduct function) transcripts. [file mmc9.pdf]

Table S8. Subset of transcripts derived from DaMiRseq R package, that could be used to separate trophectoderm samples by maternal age. Related to Figure 4 and STAR methods. RefSeq ID and associated gene names are displayed per subset of 290 (FSelect function) and 58 (FReduct function) transcripts.

refSeq (290 transcripts)	gene name	refSeq (290 transcripts)	gene name	refSeq (290 transcripts)	gene name	refSeq (58 transcripts)	gene name
NM_000041	APOE	NM_001302623	DHX8	NM_004508	IDI1	NM_000104	CYP1B1
NM_000104	CYP1B1	NM_001302688	APOE	NM_004525	LRP2	NM_000915	OXT
NM_000274	OAT	NM_001302689	APOE	NM_004748	CCPG1	NM_001005374	LRSAM1
NM_000413	HSD17B1	NM_001302690	APOE	NM_004827	ABCG2	NM_001039361	PRAMEF10
NM_000499	CYP1A1	NM_001302691	APOE	NM_005035	POLRMT	NM_001040663	GAGE1
NM_000560	CD53	NM_001303253	ACAT2	NM_005100	AKAP12	NM_001130964	PLCD1
NM_000781	CYP11A1	NM_001316964	REEP4	NM_005542	INSIG1	NM_001145140	CXorf49
NM_000786	CYP51A1	NM_001316965	REEP4	NM_005656	TMPRSS2	NM_001146109	PTGR1
NM_000915	OXT	NM_001317955	IDI1	NM_005891	ACAT2	NM_001174160	SH2D4A
NM_001002264	EPSTI1	NM_001317956	IDI1	NM_005983	SKP2	NM_001193544	ANXA6
NM_001005374	LRSAM1	NM_001317957	IDI1	NM_006184	NUCB1	NM_001197	BIK
NM_001017369	MSMO1	NM_001319075	B3GNT2	NM_006225	PLCD1	NM_001286679	LARP6
NM_001032396	PJA1	NM_001319216	CYP1A1	NM_006404	PROCR	NM_001316965	REEP4
NM_001039361	PRAMEF10	NM_001319217	CYP1A1	NM_006424	SLC34A2	NM_001319217	CYP1A1
NM_001040033	CD53	NM_001320329	NDRG2	NM_006435	IFITM2	NM_001322220	DHX8
NM_001040663	GAGE1	NM_001320638	CD53	NM_006500	MCAM	NM_001322967	OAT
NM_001042632	SNX21	NM_001320996	FABP3	NM_006558	KHDRBS3	NM_001324224	HMGCS1
NM_001042633	SNX21	NM_001321463	NCLN	NM_006577	B3GNT2	NM_001348989	ABCG2
NM_001076552	ACSS2	NM_001322218	DHX8	NM_006745	MSMO1	NM_001360	DHCR7
NM_001077494	NFKB2	NM_001322219	DHX8	NM_006868	RAB31	NM_001424	EMP2
NM_001079815	TMEM52B	NM_001322220	DHX8	NM_006927	ST3GAL2	NM_001457	FLNB
NM_001079862	DBI	NM_001322934	NFKB2	NM_012212	PTGR1	NM_001512	GSTA4
NM_001079863	DBI	NM_001322935	NFKB2	NM_012307	EPB41L3	NM_001975	ENO2
NM_001098272	HMGCS1	NM_001322965	OAT	NM_014365	HSPB8	NM_002490	NDUFA6
NM_001099773	CYP11A1	NM_001322966	OAT	NM_014573	TMEM97	NM_002502	NFKB2
NM_001111045	CCNA1	NM_001322967	OAT	NM_015687	FILIP1	NM_003129	SQLE
NM_001111046	CCNA1	NM_001322968	OAT	NM_015908	SRRT	NM_003818	CDS2
NM_001111047	CCNA1	NM_001322969	OAT	NM_015925	LSR	NM_003979	GPRC5A
NM_001127255	NLRP7	NM_001322970	OAT	NM_016084	RASD1	NM_004165	RRAD
NM_001128850	RRAD	NM_001322971	OAT	NM_016250	NDRG2	NM_004477	FRG1
NM_001128852	SRRT	NM_001322974	OAT	NM_016619	PLAC8	NM_004525	LRP2
NM_001128853	SRRT	NM_001323067	PFKP	NM_017826	SOHLH2	NM_005035	POLRMT

NM_001128854	SRRT	NM_001323068	PFKP	NM_018030	OSBPL1A	NM_005656	TMPRSS2
NM_001130715	PLAC8	NM_001323069	PFKP	NM_018641	CHST12	NM_006404	PROCR
NM_001130716	PLAC8	NM_001323070	PFKP	NM_018677	ACSS2	NM_006424	SLC34A2
NM_001130964	PLCD1	NM_001323071	PFKP	NM_019058	DDIT4	NM_006435	IFITM2
NM_001135099	TMPRSS2	NM_001323072	PFKP	NM_019079	L1TD1	NM_006500	MCAM
NM_001142776	CHAC1	NM_001323073	PFKP	NM_020166	MCCC1	NM_006577	B3GNT2
NM_001145139	CXorf49B	NM_001323074	PFKP	NM_020170	NCLN	NM_006868	RAB31
NM_001145140	CXorf49	NM_001324219	HMGCS1	NM_020548	DBI	NM_006927	ST3GAL2
NM_001145160	TPM4	NM_001324220	HMGCS1	NM_020739	CCPG1	NM_014365	HSPB8
NM_001146108	PTGR1	NM_001324222	HMGCS1	NM_020975	RET	NM_015687	FILIP1
NM_001146109	PTGR1	NM_001324223	HMGCS1	NM_021034	IFITM3	NM_018677	ACSS2
NM_001155	ANXA6	NM_001324224	HMGCS1	NM_021076	NEFH	NM_019058	DDIT4
NM_001161342	TMEM171	NM_001330219	HSD17B1	NM_021158	TRIB3	NM_020975	RET
NM_001163817	DHCR7	NM_001330543	EPSTI1	NM_022071	SH2D4A	NM_021076	NEFH
NM_001164317	FLNB	NM_001330557	EPB41L3	NM_022652	DUSP6	NM_021158	TRIB3
NM_001164318	FLNB	NM_001330663	HMGCS1	NM_024078	NOC4L	NM_022652	DUSP6
NM_001164319	FLNB	NM_001331228	EPSTI1	NM_024111	CHAC1	NM_024078	NOC4L
NM_001164835	L1TD1	NM_001343	DAB2	NM_024605	ARHGAP10	NM_024605	ARHGAP10
NM_001171814	OAT	NM_001345944	PFKP	NM_024954	UBTD1	NM_025107	MYCT1
NM_001172632	OLR1	NM_001346590	INSIG1	NM_025107	MYCT1	NM_025130	HKDC1
NM_001172633	OLR1	NM_001346591	INSIG1	NM_025130	HKDC1	NM_031308	EPPK1
NM_001174159	SH2D4A	NM_001346592	INSIG1	NM_025232	REEP4	NM_031459	SESN2
NM_001174160	SH2D4A	NM_001346593	INSIG1	NM_031308	EPPK1	NM_145119	PJA1
NM_001177998	SLC34A2	NM_001346594	INSIG1	NM_031459	SESN2	NM_153022	TMEM52B
NM_001177999	SLC34A2	NM_001348985	ABCG2	NM_032711	MAFG	NM_182810	ATF4
NM_001178017	DBI	NM_001348986	ABCG2	NM_033138	CALD1	NM_206828	NLRP7
NM_001178041	DBI	NM_001348987	ABCG2	NM_033139	CALD1		
NM_001178042	DBI	NM_001348988	ABCG2	NM_033140	CALD1		
NM_001178043	DBI	NM_001348989	ABCG2	NM_033157	CALD1		
NM_001188	BAK1	NM_001352432	DBI	NM_033255	EPSTI1		
NM_001193544	ANXA6	NM_001354558	NDRG2	NM_033421	SNX21		
NM_001197	BIK	NM_001354559	NDRG2	NM_052845	MMAB		
NM_001198910	CCDC169-SOHLH2	NM_001354560	NDRG2	NM_080597	OSBPL1A		
NM_001199989	RASD1	NM_001354561	NDRG2	NM_139176	NLRP7		
NM_001204450	CCPG1	NM_001354562	NDRG2	NM_144497	AKAP12		
NM_001204451	CCPG1	NM_001354564	NDRG2	NM_145119	PJA1		
NM_001242339	PFKP	NM_001354565	NDRG2	NM_147161	ACOT11		
NM_001242393	ACSS2	NM_001354566	NDRG2	NM_152897	SNX21		
NM_001243120	SKP2	NM_001354567	NDRG2	NM_153022	TMEM52B		
NM_001243794	CHST12	NM_001354568	NDRG2	NM_153256	PROSER2		
NM_001243795	CHST12	NM_001354569	NDRG2	NM_173490	TMEM171		

NM_001244871	DAB2	NM_001354570	NDRG2	NM_173648	CCDC141		
NM_001257386	ABCG2	NM_001360	DHCR7	NM_176782	FAM151A		
NM_001260489	LSR	NM_001424	EMP2	NM_182810	ATF4		
NM_001260490	LSR	NM_001457	FLNB	NM_198336	INSIG1		
NM_001261403	NFKB2	NM_001512	GSTA4	NM_198337	INSIG1		
NM_001281533	EPB41L3	NM_001675	ATF4	NM_201535	NDRG2		
NM_001281534	EPB41L3	NM_001946	DUSP6	NM_201536	NDRG2		
NM_001281535	EPB41L3	NM_001975	ENO2	NM_201537	NDRG2		
NM_001282211	NDRG2	NM_002087	GRN	NM_201538	NDRG2		
NM_001282212	NDRG2	NM_002130	HMGCS1	NM_201539	NDRG2		
NM_001282213	NDRG2	NM_002337	LRPAP1	NM_201540	NDRG2		
NM_001282214	NDRG2	NM_002359	MAFG	NM_201541	NDRG2		
NM_001282215	NDRG2	NM_002490	NDUFA6	NM_205834	LSR		
NM_001282216	NDRG2	NM_002502	NFKB2	NM_205835	LSR		
NM_001282633	DBI	NM_002543	OLR1	NM_206828	NLRP7		
NM_001282634	DBI	NM_002627	PFKP				
NM_001282635	DBI	NM_003129	SQLE				
NM_001282636	DBI	NM_003290	TPM4				
NM_001286679	LARP6	NM_003641	IFITM1				
NM_001288724	NFKB2	NM_003714	STC2				
NM_001289987	FILIP1	NM_003818	CDS2				
NM_001293273	MCCC1	NM_003914	CCNA1				
NM_001300866	FILIP1	NM_003979	GPRC5A				
NM_001301188	TRIB3	NM_004102	FABP3				
NM_001301190	TRIB3	NM_004165	RRAD				
NM_001301193	TRIB3	NM_004173	SLC7A4				
NM_001301196	TRIB3	NM_004342	CALD1				
NM_001301201	TRIB3	NM_004477	FRG1				
