# Supplementary material for: The effects of aging on molecular modulators of human embryo implantation
Source: iScience. 2021 Jun 19;24(7):102751. doi: 10.1016/j.isci.2021.102751 (PMC8271113; doi:10.1016/j.isci.2021.102751)
Supplement: Table S9. Report of trophectoderm genes significantly more highly expressed in young maternal age (YMA) women, along with corresponding knockout mouse studies,related to Figure 4 [file mmc10.pdf]

Table S9. Report of trophectoderm genes significantly more highly expressed in young maternal age (YMA) women, along with corresponding knockout mouse studies. Related to Figure 4.

Entrez ID	Gene name	Knockout studies/phenotypes
39	acetyl-CoA acetyltransferase 2(ACAT2)	Fertile and viable mice with abnormal embryo size (day 18.5) (Dickinson et al., 2016, JacksonLaboratory).
345	apolipoprotein C3(APOC3)	Lower cholesterol levels in blood compared with the control mice (JacksonLaboratory).
1601	DAB2, clathrin adaptor protein(DAB2)	Repression in the mouse embryonic development prior to gastrulation stage (Morris et al., 2002). Similar to lack of TGF-beta and Nodal pathway molecules phenotype (JacksonLaboratory).
1622	diazepam binding inhibitor, acyl-CoA binding protein(DBI)	Inhibition of early mouse embryo development at the morula stage (approximately 2,5 days) (JacksonLaboratory). Repression in the mouse embryonic development prior to gastrulation stage (Morris et al., 2002). Similar to lack of TGF-beta and Nodal pathway molecules phenotype (JacksonLaboratory).
1892	enoyl-CoA hydratase, short chain 1(ECHS1)	Inhibition of early mouse embryo development at the morula stage (approximately 2,5 days) (JacksonLaboratory).
2879	glutathione peroxidase 4(GPX4)	GPX4-knockout mice did not survive beyond E8.5 day (or 16 days in human) (Imai and Nakagawa, 2003, Yant et al., 2003). Low expression leads to DNA fragmentation and programmed cell death (Imai and Nakagawa, 2003, Borchert et al., 2006).
3094	histidine triad nucleotide binding protein 1(HINT1)	Sensitivity to carcinogens and increased tumorigenesis, fibroblasts with lack of Hint1 expression showed problems in DNA repair and indefinite growth. (JacksonLaboratory)

4036	LDL receptor related protein 2(LRP2)	LRP2-null mice phenotype shows increased perinatal lethality with decreased embryo size at E9.5 and increased levels of apoptosis (E9.5) in areas corresponding to neural crest (JacksonLaboratory).
7001	peroxiredoxin 2(PRDX2)	PRDX2-null mice increase programmed cell death and inhibits normal trophectoderm development, that could be involved with the accumulation of reactive oxygen species (ROS). Significantly lower PRDX2 expression has been associated with spontaneous abortions during the first trimester (Wu et al., 2017).
5134	programmed cell death 2(PDCD2)	Allows embryo implantation but not further embryo development. Inner cell mass knockout cells did not proliferate in vitro (Mu et al., 2010).
79971	wntless Wnt ligand secretion mediator(WLS)	WLS-null mouse phenotypes are lethal between E4.5 and E8 (or E9) developmental stage, which corresponds to stages between somite and placenta formation in human (organogenesis). (JacksonLaboratory)
2778	GNAS complex locus(GNAS)	GNAS gene mutation is embryonic lethal at the stage that follows the embryo implantation. (Turan and Bastepe, 2013)
