# Supplementary material for: The effects of aging on molecular modulators of human embryo implantation
Source: iScience. 2021 Jun 19;24(7):102751. doi: 10.1016/j.isci.2021.102751 (PMC8271113; doi:10.1016/j.isci.2021.102751)
Supplement: Table S10. DNA oligo sequences (primers) used to validate the RNA sequencing results by RT-qPCR,related to Figure 2 and STAR Methods — Gene names arereported together with the forward/reverse primer sequences. ACTB, GAPDH, H3F3Band HNRNPC were used as reference genes (endogenous controls). [file mmc11.pdf]

Table S10. DNA oligo sequences (primers) used to validate the RNA sequencing results by RT-qPCR. Related to Figure 2 and STAR methods. Gene names are reported together with the forward/reverse primer sequences. *ACTB*, *GAPDH*, *H3F3B* and *HNRNPC* were used as reference genes (endogenous controls).

Gene name	Primer (Forward)	Primer (Reverse)
ACTB	AAGCCACCCCACTTCTCTCT	CTATCACCTCCCCTGTGTGG
BAK1	TTCACCAAGATTGCCACCAG	ATGCTGGTAGACGTGTAGGG
BRAP	TGACTGAACTCCCCAAGTGC	GGCAAACAGGACACGTGGTA
CCPG1	CCCCGAGTGCTATACCGAAC	CAGACATCTTTCAGGTCATATGGAT
DAB2	CTAGCTATTGCAAATGAGGGAAG	GGTAATACTACTTGAACCCAGGAGCA
DBI	GCTGCGAAGTGTAGACCCTTGT	ATGGCTTGGTCTTAAGGTGCC
FABP3	CGCCTGCTCTCTTGTAGCTT	ATATGAGCGAGTGACTTCATGT
GAPDH	TTGTCAAGCTCATTTCCTGGTAT	TCTCTCTTCCTCTTGTGCTCTTG
H3F3B	TGCTGGTTTTTCGCTCGTCG	GCCATTTTCTTTCACCCAACGC
HNRNPC	GCCAGCAACGTTACCAACAA	TGAACAGAGCAGCCCACAAT
IFITM3	CTGGGCTTCATAGCATTCGCCT	AGATGTTCAGGCACTTGGCGGT
ZFAND5	GTCCATCAGTTTCTCAGCCCA	GGCAGTCAAACCCTGTAAGAC
